# Synthesis of Polyfluorinated Aromatic Selenide-Modified Polysiloxanes: Enhanced Thermal Stability, Hydrophobicity, and Noncovalent Modification Potential

**DOI:** 10.3390/polym17202729

**Published:** 2025-10-11

**Authors:** Kristina A. Lotsman, Sofia S. Filippova, Vadim Yu. Kukushkin, Regina M. Islamova

**Affiliations:** Department of Physical Organic Chemistry, Institute of Chemistry, Saint Petersburg State University, Universitetskaya Nab. 7/9, Saint Petersburg 199034, Russia; k.lotsman@spbu.ru (K.A.L.); s.filippova@spbu.ru (S.S.F.); v.kukushkin@spbu.ru (V.Y.K.)

**Keywords:** polysiloxanes, selenium, polyfluoroaromatic compounds, noncovalent interactions, thermal stability, hydrophobicity

## Abstract

Polysiloxanes are unique polymers used in medicine and materials science and are ideal for various modifications. Classic functionalization methods involve a covalent approach, but finer tuning of the properties of the final polymers can also be achieved through sub-sequent noncovalent modifications. This study introduces a fundamentally new approach to polysiloxane functionalization by incorporating cooperative noncovalent interaction centers: selenium-based chalcogen bonding donors and polyfluoroaromatic π-hole acceptors into a single polymer platform. We developed an efficient nucleophilic substitution strategy using poly((3-chloropropyl)methylsiloxane) as a platform for introducing Se-containing groups with polyfluoroaromatic substituents. Three synthetic approaches were evaluated; only direct modification of **Cl-PMS-2** proved successful, avoiding catalyst poisoning and crosslinking issues. The optimized methodology utilizes mild conditions and achieved high substitution degrees (74–98%) with isolated yields of 60–79%. Comprehensive characterization using ^1^H, ^13^C, ^19^F, ^77^Se, and ^29^Si NMR, TGA, and contact angle measurements revealed significantly enhanced properties. Modified polysiloxanes demonstrated improved thermal stability (up to 37 °C higher decomposition temperatures, 50–60 °C shifts in DTG maxima) and increased hydrophobicity (water contact angles from 69° to 102°). These systems potentially enable chalcogen bonding and arene–perfluoroarene interactions, providing foundations for materials with applications in biomedicine, electronics, and protective coatings. This dual-functionality approach opens pathways toward adaptive materials whose properties can be tuned through supramolecular modification while maintaining the inherent advantages of polysiloxane platforms—flexibility, biocompatibility, and chemical inertness.

## 1. Introduction

Polysiloxanes represent one of the most important classes of synthetic polymers, finding widespread applications across diverse fields ranging from medicine to aerospace engineering due to their unique combination of physical properties [1,2,3,4]. In these polymers, the silicon-oxygen backbone provides high polymer chain flexibility, thermal stability, chemical inertness, and biocompatibility. However, to expand the application scope of polysiloxanes, additional modification of their properties is often required, particularly enhancement of thermal stability and hydrophobicity.

Chemical modification of polymeric materials can be accomplished through two fundamentally different approaches. The covalent approach is based on post-functionalization [5,6,7] and involves the introduction of functional groups into the polymer structure through the formation of strong covalent bonds [8,9]. This method ensures stable attachment of modifying fragments and allows precise control over the degree of functionalization and distribution of functional groups along the polymer chain. The alternative supramolecular approach is based on the use of noncovalent interactions between specially introduced centers capable of directional weak bonds [10,11,12,13,14,15,16,17,18]. Supramolecular modification enables the creation of adaptive and responsive materials whose properties can change in response to external stimuli due to the reversibility of noncovalent interactions. The most effective approach involves combining both strategies, where covalently attached functional groups are capable of participating in cooperative noncovalent interactions of various types.

Within the covalent approach, traditional methods for enhancing polysiloxane thermal stability include the introduction of aromatic fragments into side chains or the main polymer backbone [19,20,21]. Aromatic groups act as thermal stabilizers, slowing depolymerization and oxidative degradation processes at elevated temperatures. Concurrently, the introduction of fluorine-containing substituents is a recognized method for enhancing both thermal stability and hydrophobicity of polymeric materials [22,23,24], in particular polysiloxanes [25,26,27]. Additionally, the incorporation of fluorinated fragments into polymers is utilized in medicine [28,29], gas separation [30], and organic electronics [31].

Polyfluoroaromatic fragments are of particular interest for implementing the supramolecular approach, as they combine the advantages of covalent modification with noncovalent interaction capabilities. Perfluorinated aromatic rings possess enhanced electron deficiency compared to conventional aromatic systems, opening possibilities for specific noncovalent interactions [32]. In particular, arene–perfluoroarene systems are capable of effective quadrupolar π_hole_–π interactions [33,34,35], which can influence polymer chain packing and, consequently, the macroscopic properties of materials. Furthermore, perfluorinated aromatic rings are strong electron-withdrawing substituents that can induce σ-hole interactions on adjacent easily polarizable atoms [36], such as halogens [15,37,38,39] or chalcogens [40,41,42].

The implementation of cooperative combinations of covalent and supramolecular approaches in polymers can be achieved, as we envision, through the introduction of chalcogen centers, particularly selenium, which after covalent attachment in the polymer structure can serve as σ-hole donors. Selenium occupies an intermediate position between sulfur and tellurium in the periodic table, demonstrating an optimal combination of atomic size, polarizability, and ability to form directional chalcogen bonds.

**Figure 1:**
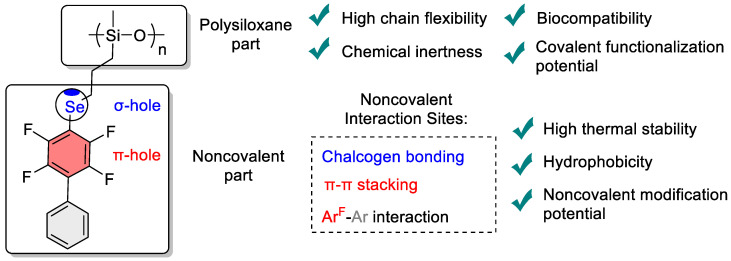
Cooperative combination of covalent and supramolecular approaches for polysiloxane modification studied in this work.

The combination of selenium σ-hole centers with polyfluoroaromatic π-systems within a single polymeric material presents particular attractiveness from the perspective of creating cooperative noncovalent interactions (Figure 1). Such hybrid systems are potentially capable of simultaneous participation in chalcogen bonds through selenium centers and in arene–perfluoroarene interactions through aromatic fragments. The cooperative action of these interactions may lead to enhanced thermal stability, and increased hydrophobicity of polymeric materials.

The polysiloxane platform represents an ideal foundation for covalent attachment of functional groups containing selenium centers and polyfluoroaromatic fragments. Well-developed methods for polysiloxane functionalization, including hydrosilylation [43,44], azide–alkyne cycloaddition reactions [45,46], and nucleophilic substitution [47], allow control over the degree of functionalization and spatial arrangement of active centers. The flexibility of the siloxane backbone provides the necessary mobility of functional groups for optimizing intermolecular interactions.

Despite the potential advantages of introducing polyfluoroaromatic selenides into polysiloxanes, systematic studies of the synthesis of such systems remain unexplored. Consequently, the main research directions should include the development of efficient synthetic approaches that would achieve high degrees of functionalization while ensuring stability of the resulting materials during storage and operation.

The primary objective of this study is to create an effective methodology for introducing selenium-containing groups with various polyfluoroaromatic substituents into the polysiloxane matrix and to investigate the influence of such modification on the thermal and hydrophobic properties of the resulting materials.

## 2. Materials and Methods

### 2.1. Materials

2,3,4,5,6-Pentafluorobiphenyl (97%), hexafluorobenzene (99%), octafluorotoluene (99%) were purchased from PiM Invest (PiM Invest, Moscow, Russia). Allyl chloride (98%) and allyl bromide (97%) were purchased from Merck KGaA (St. Louis, MO, USA). Polymethylhydrosiloxane (**PMHS**, the number average molecular weight M_n_ = 3200, viscosity 12–45 cSt), platinum (0)-1,3-divinyl-1,1,3,3-tetramethyldisiloxane complex solution 0.1 M in xylene were purchased from Abcr GmbH (Abcr GmbH, Karlsruhe, Germany). The initial perfluorinated substrate ***p*-CF_3_Ph^F^Ph^F^** was prepared from perfluorobenzene, zinc and perfluorotoluene according to the known procedure [48]. Sodium selenide was synthesized from sodium and selenium powder by the previously published method [49], then all its subsequent handling and storage were conducted under an inert atmosphere in a glovebox. Poly((3-chloropropyl)methylsiloxane) (**Cl-PMS-2**) was synthesized according to the previously published method by anionic ring-opening polymerization [47]. GPC analysis revealed that **Cl-PMS-2** exhibited a bimodal molecular weight distribution consisting of a higher molecular weight fraction (Mₙ = 6603 Da, *Đ* = 1.32) and an oligomeric fraction (Mₙ = 536 Da, *Đ* = 1.22). Toluene was freshly distilled over Na/benzophenone under argon before usage. DMF was distilled over CaH_2_ (60 °C, 12 torr) before usage.

### 2.2. Methods

The NMR spectra were recorded on a Bruker Avance III 400 NMR spectrometer (Bruker, Billerica, MA, USA) at 25 °C at 400 MHz or 500 MHz (for ^1^H NMR spectra), 101 MHz or 126 MHz (for ^13^C{^1^H} NMR spectra), 376 MHz (for ^19^F{^1^H} NMR spectra), 76 MHz or 95 MHz (for ^77^Se{^1^H} NMR spectra), 79 MHz or 99 MHz (for ^29^Si{^1^H} NMR spectra). Chemical shifts are given in *δ* values [ppm] referenced to the residual signals of non-deuterated solvent (CHCl_3_): *δ* 7.26 ppm (^1^H), 77.2 ppm (^13^C{^1^H}); CFCl_3_ δ 0.0 ppm (^19^F{^1^H}) or (CH_3_)_2_Se δ 0.0 ppm (^77^Se{^1^H}). The data were processed using MestReNova (version 6.0.2) desktop NMR data processing software.

GC-MS analysis was performed on a Shimadzu GCMSQP2010 SE instrument (Shimadzu, Kyoto, Japan). An OPTIMA1 column with 0.35 μm film thickness (25 m × 0.32 mm) was used for separation. Split injection was used, and the carrier gas was helium at a flow rate of 27.2 mL/min. The MS detector parameters were transferred line temperature 250 °C, electron energy 70 eV. The oven program mode is holding at a temperature of 60 °C for 2 min, then heating from 60 to 250 °C at a rate of 40 °C/min and further holding at a temperature of 250 °C.

Single crystals of **Allyl-Se-Ph^F^Ph** and **Allyl-S-Ph^F^Ph** were studied on a XtaLAB Synergy, Single source at home/near, HyPix (Cu K*α λ* = 1.54184 Å). The crystals were kept at 100 K during data collection. The CrysAlisPro (version 171.41.104a) [50] software package was used for cell refinements and data reductions. All structures were solved in OLEX2 program (version 1.5) [51] by direct methods using SHELXT (version 2019) [52] and refined against *F*^2^ using SHELXL (version 2019) [53]. All non-hydrogen atoms were refined with individual anisotropic displacement parameters. Crystal data, data collection and structure refinement details are summarized in Table S1. The structures have been deposited at the Cambridge Crystallographic Data Center with the reference CCDC numbers 2492181–2492182; they also contain the supplementary crystallographic data. These data can be obtained free of charge from the CCDC via http://www.ccdc.cam.ac.uk/data_request/cif (accessed on 9 October 2025).

Thermogravimetric (TG) measurements of polymers were performed on a NETZSCH TG 209F1 Libra TGA209F1D0024 (NETZSCH Group, Selb, Bavaria) analyzer in the inert (argon) atmosphere. The samples were heated from 40 °C to 1050 °C at a heating rate of 10 °C/min.

Molar masses and polydispersity indices of the polymers were determined by gel permeation chromatography (GPC) in tetrahydrofuran (THF) at 40 °C with a flow rate of 1.0 mL/min, using polystyrene standards for calibration. The GPC analysis was performed using a Shimadzu LC-20 modular system (Shimadzu, Kyoto, Japan) equipped with a GPC Column MZ-Gel SDplus 10E3A (8 mm × 300 mm) and Shimadzu RID-20A differential refractive index detector (Shimadzu, Kyoto, Japan). The analysis was conducted using two columns: 15 kDa (500 Å) and 75 kDa (1000 Å). System calibration was performed with commercially available narrow molecular-weight-distribution polystyrene standards ranging from 0.5 kDa to 1000 kDa (Polymer Laboratories, Long Beach, CA, USA). Chromatographic data were processed using Shimadzu LC solution software (version 5.90). Prior to analysis, polymer samples were filtered through a polyvinylidene difluoride (PVDF) filter (0.45 μm, 13 mm) and dissolved in THF at a concentration of 2 mg/mL.

Static water contact angle measurements were performed using the sessile drop method. Polymer samples (10 mg each) were first dissolved in chloroform (200 μL) and then deposited onto glass substrates (approximately 1 × 2 cm^2^) using a spin coater (EZ4, Lebo Science, Jiangying, China) at 2000 rpm for 1 s. Once the polymer films were prepared, distilled water droplets (10 μL) were carefully placed on the film surfaces. The samples were then photographed using a high-resolution camera system equipped with a 48-megapixel Sony IMX-803 sensor and f/1.78 aperture. To ensure measurement accuracy, multiple films were prepared for each polymer, with water droplets positioned at different locations across each sample (3 droplets per film, on 3 independent films). Contact angles were subsequently analyzed and measured using Adobe Photoshop 2024 software (version 25.6.0).

### 2.3. Synthesis of Allyl Selenide of Ph^F^Ph (**Allyl-Se-Ph^F^Ph**)

2,3,4,5,6-Pentafluorobiphenyl (732 mg, 3 mmol) was placed in a 25 mL flask, and the flask was transferred to a glovebox where DMF (9 mL) and Na_2_Se (413 mg, 3.3 mmol) were slowly added. During this process, heating of the reaction mixture and a color change to dark yellow were observed. The flask was sealed and removed from the glovebox. The reaction mixture was sonicated for 1 min and then stirred at 50 °C for 10 min. Subsequently, allyl bromide (436 mg, 3.6 mmol) was added under argon, and the yellow color of the solution disappeared. The reaction mixture was stirred at room temperature for an additional 10 min, after which it was poured into saturated aqueous NH_4_Cl solution (20 mL) and extracted with diethyl ether (3 × 20 mL). The organic layer was washed with brine (3 × 20 mL), dried over MgSO_4_, and filtered. The solvents were removed via rotary evaporation at 40 °C. The resulting product was purified by column chromatography on silica gel using hexane as the eluent. Yield is 855 mg (82%); white powder. ^1^H NMR (400 MHz, CDCl_3_) δ 7.64–7.34 (m, 5H), 5.93 (ddt, *J* = 17.4, 10.0, 7.7 Hz, 1H), 5.03 (dd, *J* = 16.9, 1.4 Hz, 1H), 4.97 (dd, *J* = 9.9, 1.4 Hz, 1H), 3.65 (d, *J* = 7.7 Hz, 2H). ^19^F{^1^H} NMR (376 MHz, CDCl_3_) δ from −128.14 to −128.68 (m, 2F), from −142.97 to −143.57 (m, 2F). ^13^C{^1^H} NMR (126 MHz, CDCl_3_) δ 147.3 (ddm, *J* = 242.1, 14.8 Hz), 143.8 (ddm, *J* = 250.1, 15.8 Hz), 133.7, 130.2, 129.3, 128.7, 127.7−127.5 (m), 121.1 (t, *J* = 17.0 Hz), 118.3, 106.4 (t, *J* = 25.0 Hz), 30.6 (t, *J* = 2.8 Hz). ^77^Se{^1^H} NMR (95 MHz, CDCl_3_) δ 215.1 (t, *J* = 13.6 Hz). GC-MS/EI (ethyl acetate, 5.4 min) *m*/*z*: 346 [M]^+·^.

### 2.4. Synthesis of Allyl Sulfide of Ph^F^Ph (**Allyl-S-Ph^F^Ph**)

2,3,4,5,6-Pentafluorobiphenyl (244 mg, 1 mmol) and DMF (3 mL) were placed in a 10 mL flask, whereupon Na_2_S (156 mg, 2 mmol) were slowly added under argon. The flask was sealed and the reaction mixture was treated with ultrasound for 1 min and then stirred at 50 °C for 10 min. After this, allyl bromide (180 mg, 1.5 mmol) was added under argon, and the yellow color of the solution disappeared. The reaction mixture was additionally stirred at room temperature for 10 min and then it was poured into a saturated NH_4_Cl solution (20 mL) and extracted with diethyl ether (3 × 20 mL). The organic layer was washed with brine (3 × 20 mL), dried over MgSO_4_, and separated by filtrations; the solvents were removed via rotary evaporation at 40 °C. The resulting product was purified by column chromatography on silica gel (hexane–ethyl acetate 50:1, *v*/*v*). Yield is 282 mg (95%); white powder. ^1^H NMR (400 MHz, CDCl_3_) δ 7.58–7.39 (m, 5H), 5.85 (ddt, *J* = 17.0, 9.9, 7.3 Hz, 1H), 5.20–4.92 (m, 2H), 3.59 (d, *J* = 7.3 Hz, 2H). ^19^F{^1^H} NMR (376 MHz, CDCl_3_) δ from −133.43 to −134.36 (m, 2F), from −143.26 to −144.21 (m, 2F). ^13^C{^1^H} NMR (101 MHz, CDCl_3_) δ 147.5 (ddm, *J* = 245.1, 14.8 Hz), 143.9 (ddm, *J* = 249.0, 15.3 Hz), 133.1, 130.2 (t, *J* = 2.2 Hz), 129.4, 128.7, 127.4, 121.0 (t, *J* = 16.8 Hz), 118.7, 112.4 (t, *J* = 20.6 Hz), 37.7 (t, *J* = 2.9 Hz). GC-MS/EI (ethyl acetate, 5.2 min) *m*/*z*: 298 [M]^+·^.

### 2.5. General Procedure of Hydrosilylation

A solution of Karstedt’s catalyst in xylene (0.1 M, 0.2 mol%, 11 µL) was placed in a 10 mL flask under argon and diluted with toluene (1 mL). Allyl chalcogenides or halides (1 mmol) were added to the resulting solution. The solution was stirred at room temperature for 1 h, and then a solution of **PMHS** (0.5 mmol, allyl:SiH ratio of 2:1) in toluene (0.5 mL) was added under argon. The flask was then sealed, and the reaction mixture was stirred at 80 °C for 72 h. During this time, a color change to light yellow (for chalcogenides) or black precipitation (for halides) was observed. Any precipitate formed was filtered off. For the chalcogenides, the solvent was removed under reduced pressure and the residue was analyzed by NMR. For the halides, the solution was partially evaporated and used in the subsequent step.

### 2.6. General Procedure for Modification of **Cl-PMS-1**

2,3,4,5,6-Pentafluorobiphenyl (31 mg, 0.13 mmol) was placed in a 5 mL flask, and the flask was transferred to a glovebox where DMF (1 mL) and Na_2_Se (17 mg, 0.14 mmol) were carefully added. During this process, heating of the reaction mixture and a color change to dark yellow were observed. The flask was sealed and removed from the glovebox. The reaction mixture was sonicated for 1 min at room temperature and then stirred at 50 °C for 10 min. Subsequently, an aliquot of polymer solution from the previous step (**Cl-PMS-1**) (0.5 mL, 0.125 mmol of polymer) was added under argon. The reaction mixture was stirred at 50 °C overnight, after which it was poured into saturated aqueous NH_4_Cl solution (20 mL) and extracted with diethyl ether (3 × 20 mL). The organic layer was washed with brine (3 × 20 mL), dried over MgSO_4_, and filtered. The solvents were removed via rotary evaporation at 40 °C. The residue was precipitated three times using a chloroform–methanol (1:20, *v*/*v*) solvent system and air-dried at room temperature.

### 2.7. General Procedure of **Cl-PMS-2** Modification

A polyfluoroaromatic compound (0.60 mmol) was placed in a 10 mL flask, and the flask was transferred to a glovebox where DMF (2 mL) and Na_2_Se (83 mg, 0.66 mmol) were slowly added. During this process, heating of the reaction mixture and a color change to dark yellow were observed. The flask was sealed and removed from the glovebox. The reaction mixture was sonicated for 1 min at room temperature and then stirred at 50 °C (for Ph^F^Ph) or room temperature (for Ph^F^CF_3_ or CF_3_Ph^F^Ph^F^) for 10 min. Subsequently, a solution of **Cl-PMS-2** (70 mg, 0.5 mmol) in THF (0.8 mL) was added under argon. The reaction mixture was stirred at room temperature (for Ph^F^CF_3_ or CF_3_Ph^F^Ph^F^) or 50 °C (for Ph^F^Ph) for 48 h, after which it was poured into saturated aqueous NH_4_Cl solution (20 mL) and extracted with diethyl ether (3 × 20 mL). The organic layer was washed with brine (3 × 20 mL), dried over MgSO_4_, and filtered. The solvents were removed by rotary evaporation at 40 °C. The resulting product was purified by flash column chromatography on silica gel using a hexane–ethyl acetate gradient (100:0 to 0:100) as the eluent.

**PhPh^F^-Se-PMS**: Yield is 162 mg (79%); Cl substitution degree: 92%; viscous yellow oil. ^1^H NMR (500 MHz, CDCl_3_) δ 7.71–7.34 (br m, 5H), 3.04 (br m, 2H), 1.92–1.64 (br m, 2H), 0.79–0.55 (br m, 2H), from 0.14 to −0.00 (br m, 3H). ^19^F{^1^H} NMR (376 MHz, CDCl_3_) δ from −128.61 to −129.10 (m, 2F), from −142.98 to −143.35 (m, 2F). ^13^C{^1^H} NMR (126 MHz, CDCl_3_) δ 147.3 (dd, *J* = 242.1, 14.5 Hz), 143.7 (ddt, *J* = 249.8, 16.3, 4.8 Hz), 130.2, 129.3, 128.7, 127.6, 120.7 (t, *J* = 16.7 Hz), 106.8 (m), 31.9 (m), 24.6 (m), 19.0–16.5 (m), from −0.4 to −0.9 (m). ^77^Se{^1^H} NMR (95 MHz, CDCl_3_) δ 177.6–173.9 (m). ^29^Si{^1^H} NMR (79 MHz, CDCl_3_) δ from −20.0 to −21.0 (m).

**CF_3_Ph^F^-Se-PMS:** Yield: 120 mg (60%); Cl substitution degree: 98%; viscous yellow oil. ^1^H NMR (500 MHz, CDCl_3_) δ 3.11 (br m, 2H), 1.81–1.61 (br m, 2H), 0.73–0.55 (br m, 2H), from 0.12 to −0.04 (br m, 3H). ^19^F{^1^H} NMR (376 MHz, CDCl_3_) δ from −54.84 to −58.23 (m, 3F), from −125.52 to −128.54 (m, 2F), from −138.96 to −142.22 (m, 2F). ^13^C{^1^H} NMR (126 MHz, CDCl_3_) δ 146.9 (d, *J* = 243.8 Hz), 143.9 (dd, *J* = 262.2, 18.6 Hz), 120.9 (q, *J* = 272.9 Hz), 114.2 (m), 109.7–108.2 (m), 31.8, 24.8–24.3 (m), 17.9, −0.5. ^77^Se{^1^H} NMR (95 MHz, CDCl_3_) δ 212.1–201.3 (m). ^29^Si{^1^H} NMR (99 MHz, CDCl_3_) δ −20.6, −21.9, from −22.6 to −23.5 (m).

**CF_3_Ph^F^Ph^F^-Se-PMS:** Yield is 190 mg (69%); Cl substitution degree: 74%; viscous yellow oil. ^1^H NMR (400 MHz, CDCl_3_) δ 3.14 (br s, 2H), 1.79 (br s, 2H), 0.81–0.53 (br m, 2H), from 0.17 to −0.10 (br m, 3H). ^19^F{^1^H} NMR (376 MHz, CDCl_3_) δ −56.63 (br s, 3F), −127.52 (br s, 2F), −135.95 (br s, 2F), −137.75 (br s, 2F), −139.33 (br s, 2F). ^13^C{^1^H} NMR (101 MHz, CDCl_3_) δ 146.9 (d, *J* = 228.2 Hz), 144.5 (dm, *J* = 256.0 Hz), 143.7 (dd, *J* = 255.6, 17.7 Hz), 120.6 (q, *J* = 272.8 Hz), 113.7–110.1 (m), 105.9–104.6 (m), 47.5, 32.0, 26.8, 24.6, 18.0, 15.1, −0.4. ^77^Se{^1^H} NMR (76 MHz, CDCl_3_) δ 195.4 (br m). ^29^Si{^1^H} NMR (79 MHz, CDCl_3_) δ from −22.2 to −22.9 (m), from −22.9 to −23.6 (m).

## 3. Results and Discussion

**Scheme 1:**
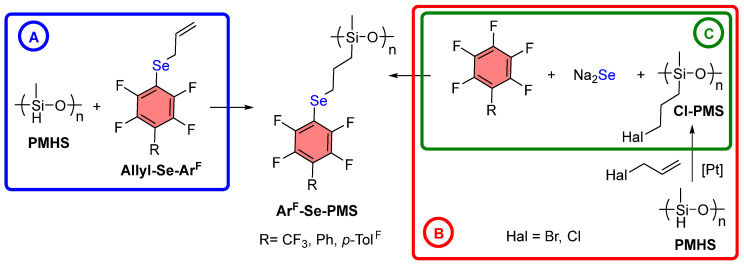
Various approaches to the synthesis of polysiloxanes containing polyfluorinated aromatic selenide fragments: from **PMHS** (**A**) direct hydrosilylation of allyl selenide and (**B**) two-stage hydrosilylation process (synthesis of **Cl-PMS-1**) followed by nucleophilic substitution reaction) and (**C**) from **Cl-PMS-2**.

Several approaches were proposed for the introduction of selenides containing polyfluorinated aromatic fragments into polysiloxanes. Since **PMHS** (Scheme 1) is one of the commercially available polysiloxanes, approach A was initially proposed, namely direct hydrosilylation between **PMHS** and allyl selenide. The subsequent approach B involved sequential hydrosilylation reactions between **PMHS** and allyl halides, aimed at obtaining a halogen-containing polymer (**Cl-PMS-1**), which would then undergo nucleophilic substitution with Na_2_Se and a polyfluoroaromatic compound. Finally, the third approach C was based on reactions with a chlorine-containing polysiloxane (**Cl-PMS-2**), previously synthesized via anionic ring-opening polymerization according to an established procedure [47].

### 3.1. Synthesis of **Ar^F^-Se-PMS** Through **PMHS** Modification

**Scheme 2:**
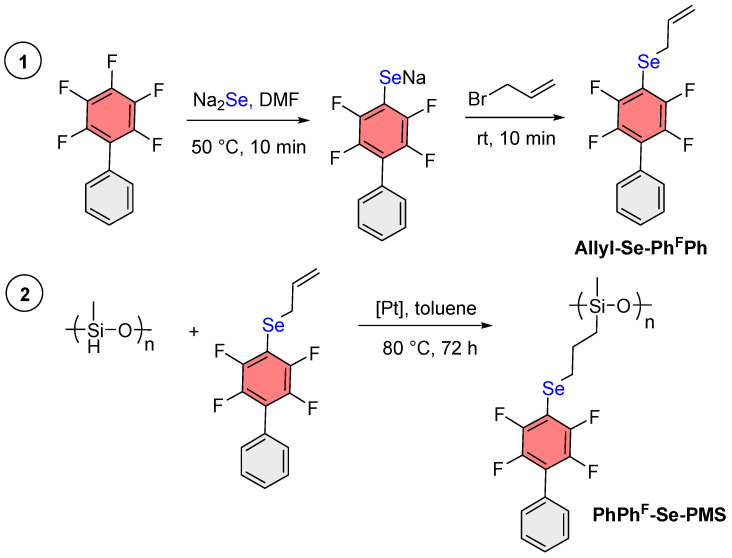
(**1**) Synthesis of **Allyl-Se-Ph^F^Ph** and (**2**) subsequent hydrosilylation with **PMHS**.

For the direct hydrosilylation reaction with **PMHS** (Scheme 1A), **Allyl-Se-Ph^F^Ph** was synthesized via nucleophilic aromatic substitution [40] from Ph^F^Ph, Na_2_Se, and allyl bromide (Scheme 2). A single crystal of this starting compound (**Allyl-Se-Ph^F^Ph)** was grown and its structure was determined by X-ray crystallography. An interesting feature of this structure is the infinite stacking arrangement arising from complementary aryl–perfluoroaryl interactions (Figures S1–S3).

Subsequently, a hydrosilylation reaction was carried out between **Allyl-Se-Ph^F^Ph** and **PMHS** using Karstedt’s catalyst (0.2 mol%) at 80 °C for 72 h [43]. Monitoring the reaction with ^1^H NMR revealed that the target product was not formed under these conditions. Apparently, the starting selenide participates in noncovalent interactions with platinum [54,55,56], which prevents the hydrosilylation reaction from proceeding.

To test this hypothesis, an analogous sulfide (**Allyl-S-Ph^F^Ph**) was synthesized. The structure of this compound was studied by X-ray crystallography and was shown to be isostructural with the corresponding selenide (Figures S4–S6). When the reaction was conducted with this sulfide, it was found that the target product was formed in 40% ^1^H NMR yield after 48 h, confirming the hypothesis of reaction inhibition in the case of selenium.

**Scheme 3:**
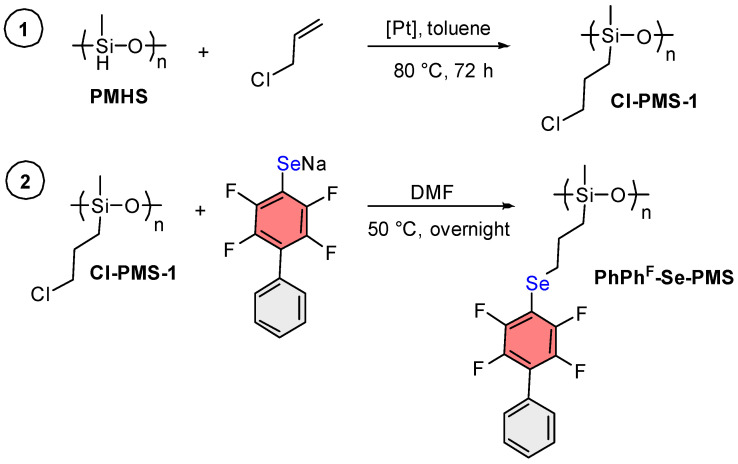
(**1**) Synthesis of **Cl-PMS-1** from **PMHS** and (**2**) subsequent nucleophilic substitution reaction.

Since direct hydrosilylation with the selenide **Allyl-Se-Ph^F^Ph** does not proceed, we turned to pathway B. According to this strategy, it was first intended to conduct a hydrosilylation reaction between **PMHS** and allyl chloride or bromide, followed by a nucleophilic substitution reaction with the resulting polymer (**Hal-PMS**), Ph^F^Ph, and Na_2_Se (as in the synthesis of starting **Allyl-Se-Ph^F^Ph**) (Scheme 3).

For allyl bromide and allyl chloride, the same hydrosilylation reaction conditions were employed as described in pathway A for **Allyl-Se-Ph^F^Ph**. However, the reaction with allyl bromide proceeded poorly, yielding a crosslinked polymer rather than the desired target product. This outcome is attributed to a competing side reaction involving propylene moiety formation [57], which can occur both prior to and following the reaction between allyl bromide and **PMHS**. The propylene moiety bound to **PMHS** is susceptible to subsequent hydrosilylation reactions, ultimately leading to crosslinked polymer formation. Alternatively, this results from **PMHS** crosslinking through Si–H interactions with formation of Si–Si groups via dehydrocoupling reaction, which are oxidized in air to Si–O–Si [58]. In the case of allyl chloride, Si–H signals were not observed in the ^1^H NMR spectrum; instead, a new signal appeared around 3.60 ppm corresponding to the Cl-CH_2_ protons of the target reaction product (**Cl-PMS-1**), while crosslinked polymer formation was not visually observed. To remove excess starting allyl chloride as well as the formed platinum black, the reaction mixture was filtered through Celite after the reaction and then concentrated under reduced pressure at 40 °C to approximately one-third of the initial volume. Upon complete solvent removal, the final polymer crosslinks (swelling in organic solvents rather than dissolving), which complicates further work with it. An aliquot was taken from the obtained **Cl-PMS-1** solution and used in the next stage of the process.

In the second stage, a reaction was carried out between **Cl-PMS-1** and PhPh^F^SeNa in DMF. ^1^H NMR monitoring showed that the reaction proceeded to 40% completion at room temperature in 8 h (substitution of Cl atoms with Se). Under optimal conditions (polymer–PhPh^F^SeNa ratio 1:1, 50 °C, overnight), the degree of Cl substitution was 85%. After isolation of this polymer and three-fold reprecipitation from a chloroform–methanol solvent system, the polymer was obtained as a beige powder in 31% yield (16 mg). After drying in air overnight, this polymer was only partially soluble in CDCl_3_, which indicated its partial crosslinking. When this reaction was conducted with PhPh^F^SNa, the target product after reprecipitation also appeared as a beige powder (21 mg, 46% yield) and also partially crosslinked after drying.

When attempting to scale up the reaction from 0.125 mmol to 1 mmol of Ph^F^Ph for the synthesis of **PhPh^F^-Se-PMS**, the yield of the target product decreased further to 15% (61 mg), while the problem of partial polymer crosslinking after drying persisted. The instability of the final polymers during air storage interfered with further characterization and application.

To determine at which stage polymer crosslinking occurred, the solvent was evaporated from the **Cl-PMS-1** solution immediately after its preparation, and the resulting polymer was analyzed by ^29^Si NMR spectroscopy. The ^29^Si NMR spectrum showed resonances in the range of −65 ppm to −68 ppm, characteristic of Si–O–Si bonds, indicating that partial crosslinking occurred specifically during the allyl chloride grafting stage. Given the storage instability of polymers obtained by this method, we subsequently abandoned this synthetic approach.

Thus, we demonstrated that direct hydrosilylation of **PMHS** with allyl chalcogenides is only possible in the case of sulfides, but with a low degree of addition (40%). An alternative method for **PMHS** modification through reaction with allyl chloride followed by interaction with Na_2_Se and Ph^F^Ph is feasible, but ultimately leads to a polymer that partially crosslinks in air during storage.

### 3.2. Synthesis of **Ar^F^-Se-PMS** from **Cl-PMS-2**

Since the reactions with **PMHS** performed unsatisfactorily, it was decided to synthesize **Cl-PMS-2** via anionic ring-opening polymerization of cyclo-oligo(3-chloropropyl)methylsiloxane in the presence of [Me_4_N]OH initiator [47]; the polymer yield was 72%.

**Scheme 4:**
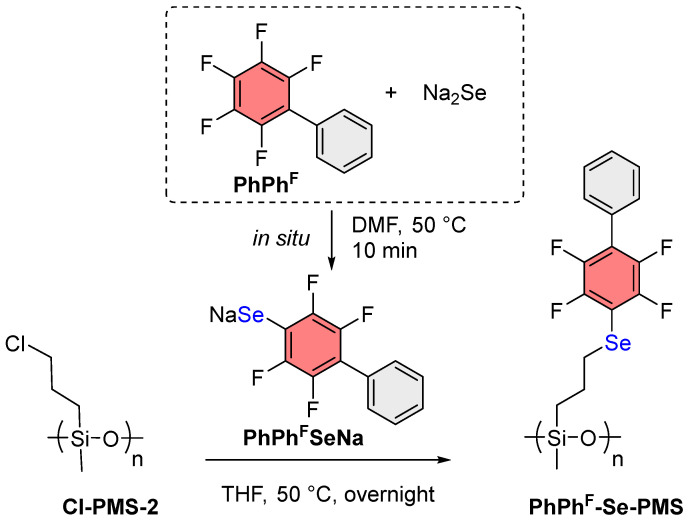
Preparation of **PhPh^F^-Se-PMS** from **Cl-PMS-2**.

The obtained **Cl-PMS-2** was subjected to nucleophilic substitution reaction with PhPh^F^SeNa (Scheme 4). According to ^1^H NMR monitoring, the degree of Cl substitution was 73% overnight. Formation of a byproduct (PhPh^F^H) was also observed. To address this issue, different reagent ratios were investigated (Table 1). Upon increasing the reagent ratio, the degree of the substitution also increased; however, the amount of reaction byproducts also increased, including the diselenide PhPh^F^SeSePh^F^Ph, which formed due to decomposition of unreacted PhPh^F^SeNa in air at room temperature during isolation of the reaction product. Thus, the optimal **Cl-PMS-2**:PhPh^F^SeNa ratio was found to be 1:1.2, at which diselenide generation was not observed and maximum Cl substitution degree of 92% was achieved. The obtained polymer after isolation was liquid, and flash column chromatography proved to be the best purification method. After purification, **PhPh^F^-Se-PMS** was obtained in 79% isolated yield.

**Scheme 5:**
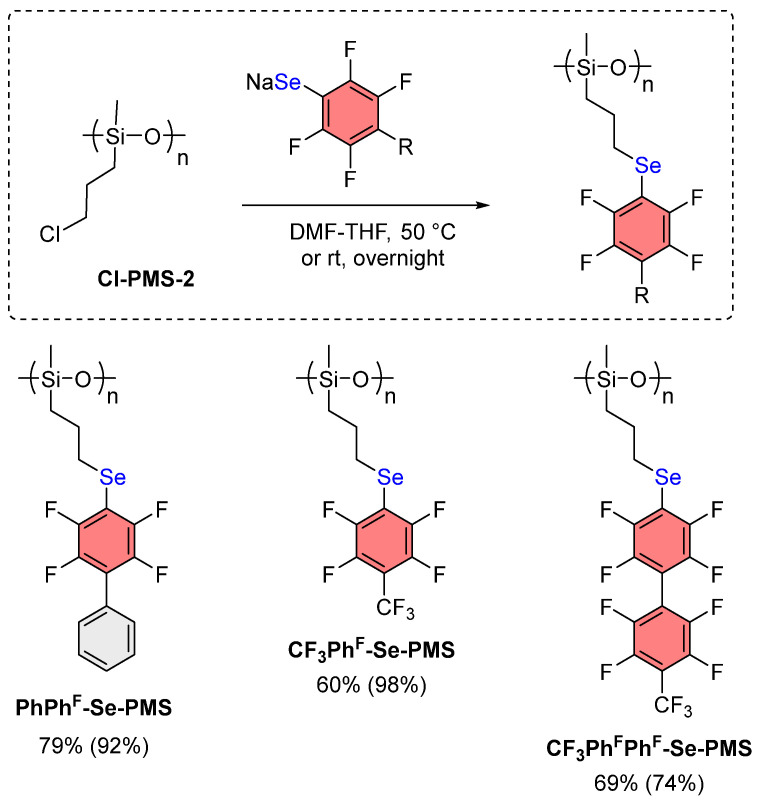
Modification of **Cl-PMS-2** with polyfluorinated selenides. Isolated yields are shown, with the ^1^H NMR-based degree of Cl substitution in the final polymer given in parentheses.

This methodology was also extended to perfluoroaromatic substrates **CF_3_Ph^F^** and ***p*-CF_3_Ph^F^Ph^F^** (Scheme 5). In the case of perfluorotoluene under the same conditions, a higher degree (98%) of Cl substitution in the final polymer was achieved, although the isolated yield was lower (60%). Meanwhile, upon introduction of more bulky fragment ***p*-CF_3_Ph^F^Ph^F^**, a decrease in the Cl substitution degree to 74% was observed, apparently due to steric hindrance. This results from repulsion between bulky perfluorinated aromatic substituents in highly substituted polymer chains.

The molecular weights of the resulting compounds were determined by GPC (Table S2, Figures S29–S32). Analysis of the GPC curves revealed that the modified polymers **CF_3_Ph^F^-Se-PMS** (M_n_ = 10040 Da, *Đ* = 1.55) and **CF_3_Ph^F^Ph^F^-Se-PMS** (M_n_ = 10420 Da, *Đ* = 1.39) exhibited molecular weights comparable to the initial **Cl-PMS-2** (M_n_ = 6600 Da, *Đ* = 1.32). The slight increase in molecular weight is consistent with the successful incorporation of bulky perfluoroaromatic substituents. Also, less intense signals corresponding to oligomeric fractions were observed for **Cl-PMS-2** (M_n_ = 540 Da, *Đ* = 1.22), **CF_3_Ph^F^-Se-PMS** (M_n_ = 350 Da, *Đ* = 1.21), **CF_3_Ph^F^Ph^F^-Se-PMS** (M_n_ = 500 Da, *Đ* = 1.38). These oligomers can be formed during the synthesis of **Cl-PMS** [59]. Notably, for **PhPh^F^-Se-PMS**, only an oligomeric fraction was identified (M_n_ = 1140 Da, *Đ* = 1.10), suggesting that the low molecular weight fraction of **Cl-PMS-2** reacted, and subsequently, this modified oligomer was isolated during purification. All obtained polymers were viscous liquids that can be stored in air at room temperature without visible changes for at least 2 months.

Thus, a method was developed for introducing polyfluorinated selenide fragments into polysiloxanes via a nucleophilic substitution reaction with **Cl-PMS-2** and Ar^F^SeNa. This approach enables Cl substitution of 74–98% with isolated yields of the target polymer of 60–79%; the reaction proceeds under mild conditions.

### 3.3. Polymer Properties

The thermal properties of the obtained polymers were studied using TGA (Table 2). Upon comparison of the 5% mass loss temperatures (T_d,5%_; Figure S33), the parent **Cl-PMS-2** exhibited the lowest thermal stability at 280 °C, which is comparable to typical polydimethylsiloxane (**PDMS**) [60,61] and commercially available Sylgard 186 [62] with an onset decomposition temperature of approximately 250 °C. For **CF_3_Ph^F^-Se-PMS**, T_d,5%_ increased to 293 °C, for **CF_3_Ph^F^Ph^F^-Se-PMS** to 314 °C, and for **PhPh^F^-Se-PMS** to 317 °C. These modifications resulted in thermal stability enhancements of 13–37 °C compared to the parent polymer, with **PhPh^F^-Se-PMS** showing the greatest improvement. A comparison with some known fluorine and phenyl containing polysiloxanes is presented in Table 2: poly(methyl(trifluoropropyl)siloxane) (**PMTFPS**), modified **P(MTFPS-co-DPS)-1** with diphenylsiloxane units [19] and phenanthrenylmethyl containing polysiloxane (**HM40_PHM20_PP40**) [20].

**Figure 2:**
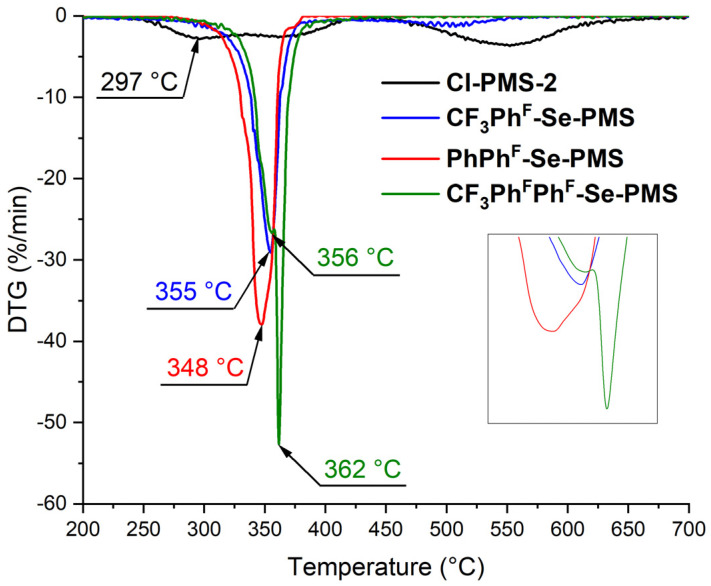
DTG comparison of the parent and modified polysiloxanes. Temperatures of the first mass loss stage are shown.

The most interesting effect of the polyfluoroaromatic selenide fragment was observed on the rate of mass loss, which was monitored using DTG (Figure 2). According to DTG analysis, the parent **Cl-PMS-2** exhibited three stages of mass loss, with the first maximum mass loss occurring at 297 °C. For **PhPh^F^-Se-PMS**, only one stage of mass loss was observed within a narrow temperature range, with the maximum shifted to 348 °C. For **CF_3_Ph^F^-Se-PMS** and **CF_3_Ph^F^Ph^F^-Se-PMS**, two stages were observed, with the maximum of the first stage further increased to 355 °C and 356 °C, respectively. These results demonstrate that incorporating fluorinated and perfluorinated aromatic rings into the polysiloxane structure substantially improves thermal stability, with DTG measurements revealing temperature enhancements of 50–60 °C. However, increasing the number of F atoms in the polymer molecules did not exert a significant influence on the thermal properties.

**Figure 3:**
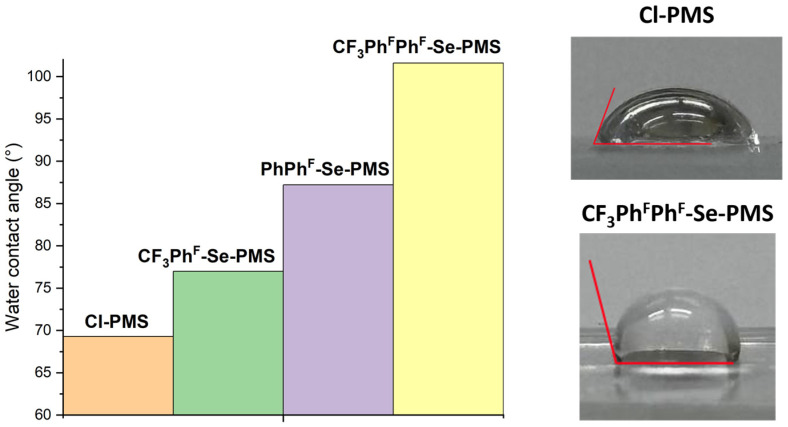
Comparison of water contact angles for the parent and modified polysiloxanes.

The hydrophobic properties of the obtained polysiloxanes were studied by water contact angle measurements. For this purpose, the polymers were deposited onto glass slides using a spin coater, and then a drop of distilled water was placed on the resulting thin polymer film (Figures S34–S37). The drop was subsequently photographed and the contact angles were determined from the photographs (Figure 3).

The water contact angle for the parent polymer was 69.3° ± 1.2°, indicating its hydrophilic nature (angles less than 90° signify hydrophilic behavior, while angles greater than 90° indicate hydrophobic properties). Upon introduction of the perfluorotolyl substituent, the water contact angle increased to 77.0° ± 5.7°, making the polymer more hydrophobic. The relatively large standard deviation in this case may be attributed to film roughness, which is commonly observed for spin-coated viscous polymers. The **PhPh^F^-Se-PMS** exhibited even greater hydrophobicity (87.2° ± 3.3°), and finally, the most hydrophobic in the series was **CF_3_Ph^F^Ph^F^-Se-PMS** (101.6° ± 2.3°). Thus, the introduction of bulkier aromatic substituents containing the highest number of fluorine atoms expectedly increased the polymer hydrophobicity. This result underscores the critical role of fluorine content in surface wettability.

**Figure 4:**
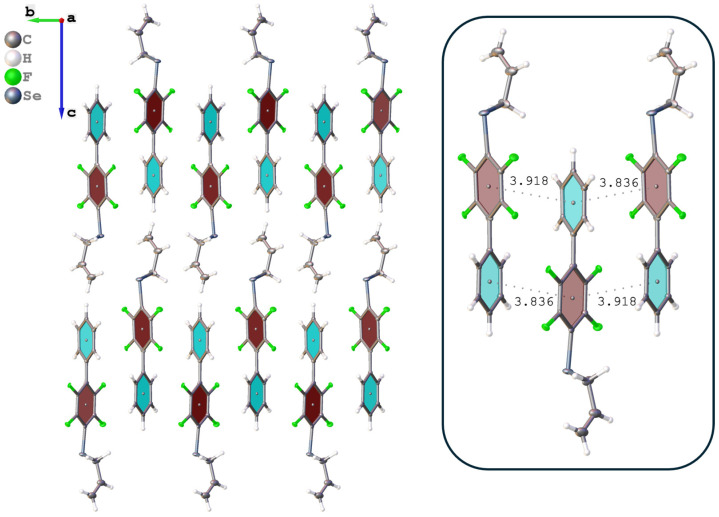
Aryl–perfluoroaryl interactions in the crystal of the **Allyl-Se-Ph^F^Ph** monomer.

An interesting property of the obtained polymeric materials is their potential for further modification through noncovalent interactions. During single-crystal X-ray diffraction analysis of **Allyl-Se-Ph^F^Ph** and **Allyl-S-Ph^F^Ph** monomers (Figures S1–S6), short contacts were observed between the planes of fluorinated and non-fluorinated aromatic rings, apparently associated with intramolecular aryl–perfluoroaryl interactions (Figure 4). It can be hypothesized that similar interactions may persist in the polymer, organizing its structure and endowing it with novel properties such as conductivity [34]. The polyfluoroaromatic selenide fragment can act as a chalcogen bond donor and also engage in π-hole interactions with other species, as previously demonstrated with monomeric systems [40,42,56,63]. These interactions can be exploited for creating sensors responsive to various anions and self-healing materials. Detailed investigation of supramolecular modification through these interactions in polymeric systems will be the subject of future research, while the present work provides a general tool for constructing such systems.

It can be concluded that the incorporation of polyfluoroaromatic selenide fragments into polysiloxanes enhances both the thermal stability and hydrophobicity of the polymers.

## 4. Conclusions

This study has successfully established an efficient and versatile synthetic methodology for introducing polyfluorinated aromatic selenide fragments into polysiloxane matrices through nucleophilic substitution reactions with **Cl-PMS-2**. The developed approach circumvents the limitations encountered with direct hydrosilylation methods, providing a reliable route to functional polymers with substitution degrees ranging from 74% to 98% and isolated yields of 60–79% under mild reaction conditions.

The resulting modified polysiloxanes demonstrate markedly enhanced material properties compared to the parent polymer. Thermal stability improvements of 13–37 °C in decomposition temperatures, coupled with substantial enhancements in decomposition behavior as evidenced by DTG analysis, highlight the beneficial impact of polyfluoroaromatic selenide incorporation. Concurrently, the systematic increase in hydrophobicity, with water contact angles progressing from 69° for the parent polymer to 102° for the most heavily fluorinated derivative, underscores the effectiveness of this modification strategy. The materials exhibit excellent storage stability under ambient conditions, maintaining their properties without degradation over extended periods.

The incorporated selenium centers and polyfluoroaromatic fragments possess σ-hole and π-hole donor capabilities, respectively, which create opportunities for cooperative noncovalent interactions including chalcogen bonding and arene–perfluoroarene interactions. Exploration of these supramolecular modification pathways will be conducted in the further development of this project to potentially unlock additional material functionalities and expand application possibilities.

The synthetic methodology, comprehensive characterization, and structure–property relationships presented herein provide a blueprint for designing next-generation functional materials with applications spanning (1) hydrophobic and thermally stable protective coatings, (2) electronic materials exploiting the unique electronic properties of selenium and fluorinated aromatics, (3) potential sensing applications based on σ-hole and π-hole interactions with specific analytes, and (4) biomedical applications leveraging biocompatibility with enhanced properties.

## Figures and Tables

**Table 1:** The effect of the reagents ratio on the Cl substitution degree and the byproducts formation.

№	Cl-PMS-2: PhPh^F^SeNa Ratio	Cl Substitution Degree, ^a^ mol%	Byproduct, ^b^ mol%	
			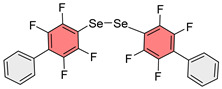 PhPh^F^Se-SePh^F^Ph	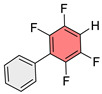 PhPh^F^H
1	1:1	73	0	23
2	1:1.3	92	3	16
3	1:1.5	90	11	29
4	1:1.7	88	15	31
5	1:1.2	92	0	23

^a^ Detected by ^1^H NMR. ^b^ Detected by ^19^F{^1^H} NMR.

**Table 2:** Comparison of thermal stability (in an inert atmosphere) of polymers synthesized in this work with known ones.

Polymer	Atmosphere	T_d,2%_, ^a^ °C	T_d,5%_, ^b^ °C	T_max%_, ^c^ °C	Residue Mass, ^d^ %	Ref.
**Cl-PMS-2**	Ar	260	280	297	22	This study
**CF_3_Ph^F^-Se-PMS**	Ar	237	293	355	10	This study
**CF_3_Ph^F^Ph^F^-Se-PMS**	Ar	271	314	356	2	This study
**PhPh^F^-Se-PMS**	Ar	301	317	348	1	This study
**Sylgard 186**	N_2_	248	–	400	≈80	[62]
**HM40_PHM20_PP40**	N_2_	220	282	–	≈30	[20]
**PMTFPS**	N_2_	250	345	404	≈2	[19]
**P(MTFPS-co-DPS)-1**	N_2_	270	366	412	≈2	[19]

^a^ 2% weight loss temperature; ^b^ 5% weight loss temperature; ^c^ temperature at maximum degradation rate; ^d^ at 800 °C and 700 °C (for **PMTFPS** and **P(MTFPS-co-DPS)-1**).

## Data Availability

The original contributions presented in this study are included in the article/Supplementary Materials. Further inquiries can be directed to the corresponding author.

## Supplementary Materials

The following supporting information can be downloaded at: https://www.mdpi.com/article/10.3390/polym17202729/s1, Table S1: Crystal data and structure refinements; Table S2: Number-average and weight-average molecular weights and dispersity of the polymers; Figure S1: The structure of **Allyl-Se-Ph^F^Ph**; Figure S2: fragment of a package in **Allyl-Se-Ph^F^Ph** crystal; Figure S3: The aryl-perfluoroaryl interaction in **Allyl-Se-Ph^F^Ph**; Figure S4: The structure of **Allyl-S-Ph^F^Ph**; Figure S5: A fragment of a package in **Allyl-S-Ph^F^Ph** crystal; Figure S6: The aryl-perfluoroaryl interaction in **Allyl-S-Ph^F^Ph**; Figure S7: ^1^H NMR (400 MHz, CDCl_3_) spectrum of **Allyl-Se-Ph^F^Ph**; Figure S8: ^19^F{**^1^**H} NMR (376 MHz, CDCl_3_) spectrum of **Allyl-Se-Ph^F^Ph**; Figure S9: ^13^C{**^1^**H} NMR (126 MHz, CDCl_3_) spectrum of **Allyl-Se-Ph^F^Ph**.; Figure S10: ^77^Se NMR (95 MHz, CDCl_3_) spectrum of **Allyl-Se-Ph^F^Ph**; Figure S11: ^1^H NMR (400 MHz, CDCl_3_) spectrum of **Allyl-S-Ph^F^Ph**; Figure S12: ^19^F{**^1^**H} NMR (376 MHz, CDCl_3_) spectrum of **Allyl-S-Ph^F^Ph**; Figure S13: ^13^C{**^1^**H} NMR (101 MHz, CDCl_3_) spectrum of **Allyl-S-Ph^F^Ph**; Figure S14: ^1^H NMR (500 MHz, CDCl_3_) spectrum of **Ph^F^Ph-Se-PMS**; Figure S15: ^19^F{**^1^**H} NMR (376 MHz, CDCl_3_) spectrum of **Ph^F^Ph-Se-PMS**; Figure S16: ^13^C{**^1^**H} NMR (126 MHz, CDCl_3_) spectrum of **Ph^F^Ph-Se-PMS**; Figure S17: ^77^Se NMR (95 MHz, CDCl_3_) spectrum of **Ph^F^Ph-Se-PMS**; Figure S18: ^29^Si NMR (79 MHz, CDCl_3_) spectrum of **Ph^F^Ph-Se-PMS**; Figure S19: ^1^H NMR (500 MHz, CDCl_3_) spectrum of **CF3Ph^F^-Se-PMS**; Figure S20: ^19^F{**^1^**H} NMR (376 MHz, CDCl_3_) spectrum of **CF3Ph^F^-Se-PMS**; Figure S21: ^13^C{**^1^**H} NMR (126 MHz, CDCl_3_) spectrum of **CF3Ph^F^-Se-PMS**; Figure S22: ^77^Se NMR (95 MHz, CDCl_3_) spectrum of **CF3Ph^F^-Se-PMS**; Figure S23: ^29^Si NMR (99 MHz, CDCl_3_) spectrum of **CF3Ph^F^-Se-PMS**; Figure S24: ^1^H NMR (400 MHz, CDCl_3_) spectrum of **CF3Ph^F^Ph^F^-Se-PMS**; Figure S25: ^19^F{**^1^**H} NMR (376 MHz, CDCl_3_) spectrum of **CF3Ph^F^Ph^F^-Se-PMS**; Figure S26: ^13^C{**^1^**H} NMR (101 MHz, CDCl_3_) spectrum of **CF3Ph^F^Ph^F^-Se-PMS**; Figure S27: ^77^Se NMR (76 MHz, CDCl_3_) spectrum of **CF3Ph^F^Ph^F^-Se-PMS**; Figure S28: ^29^Si NMR (79 MHz, CDCl_3_) spectrum of **CF3Ph^F^Ph^F^-Se-PMS**; Figure S29: GPC graphic of **Cl-PMS**; Figure S30: GPC graphic of **CF3Ph^F^-Se-PMS**; Figure S31: GPC graphic of **CF3Ph^F^Ph^F^-Se-PMS**; Figure S32: GPC graphic of **PhPh^F^-Se-PMS**; Figure S33: TGA curves of synthesized polymers. T_d,5%_: **Cl-PMS**—280 °C, **PhPh^F^-Se-PMS**—317 °C, **CF3Ph^F^-Se-PMS**—293 °C, **CF3Ph^F^Ph^F^-Se-PMS**—314 °C; Figure S34: Representative contact angles for **Cl-PMS**; Figure S35: Representative contact angles for **CF3Ph^F^-Se-PMS**; Figure S36: Representative contact angles for **PhPh^F^-Se-PMS**; Figure S37: Representative contact angles for **CF3Ph^F^Ph^F^-Se-PMS**.
