# Supplementary material for: Synthesis of Polyfluorinated Aromatic Selenide-Modified Polysiloxanes: Enhanced Thermal Stability, Hydrophobicity, and Noncovalent Modification Potential
Source: Polymers (Basel). 2025 Oct 11;17(20):2729. doi: 10.3390/polym17202729 (PMC12566975; doi:10.3390/polym17202729)
Supplement: Supplementary file 1 [file polymers-17-02729-s001.zip › polymers-3897469-supplementary.pdf]

Supporting Information

Synthesis of Polyfluorinated Aromatic Selenide-Modified Polysiloxanes: Enhanced Thermal Stability, Hydrophobicity, and Noncovalent Interaction Potential

Kristina A. Lotsman, Sofia S. Filippova, Vadim Yu. Kukushkin and Regina M. Islamova*

Saint Petersburg State University, 7/9 Universitetskaya nab., St. Petersburg, 199034, Russia
E-mail: r.islamova@spbu.ru

Contents

S1. Single-crystal X-ray structure determination 3

S2. NMR spectra 7

S3. Molecular weights of polymers 18

S4. TGA data 20

S5. Hydrophobicity test 21

S1. Single-crystal X-ray structure determination

Table S1. Crystal data and structure refinements.

	**Allyl-Se-Ph^{F}Ph**	**Allyl-S-Ph^{F}Ph**
Empirical formula	$C_{15}H_{10}F_4Se$	$C_{15}H_{10}F_4S$
Formula weight	345.19	298.29
Temperature/K	100(2)	100(2)
Crystal system	monoclinic	monoclinic
Space group	$P2_1/c$	P2/c
a/Å	5.7784(2)	7.6111(2)
b/Å	7.6103(2)	5.7555(2)
c/Å	29.2488(8)	28.9950(8)
α/°	90	90
β/°	92.243(3)	92.059(2)
γ/°	90	90
Volume/Å^3	1285.24(7)	1269.33(7)
Z	4	4
ρ_{calc}g/cm^3	1.784	1.561
μ/mm^{-1}	4.310	2.621
F(000)	680.0	608.0
Crystal size/mm^3	0.05 × 0.03 × 0.01	0.05 × 0.03 × 0.03
Radiation	Cu Kα (λ = 1.54184)	Cu Kα (λ = 1.54184)
2Θ range for data collection/°	6.048 to 139.95	6.1 to 134.92
Index ranges	$-7 \le h \le 7$, $0 \le k \le 9$, $0 \le l \le 35$	$-9 \le h \le 9$, $0 \le k \le 6$, $0 \le l \le 34$
Reflections collected	2420	2255
Independent reflections	2420 [R_{int} = ?, R_{sigma} = 0.0621]	2255 [R_{int} = ?, R_{sigma} = 0.0329]
Data/restraints/parameters	2420/48/182	2255/0/182
Goodness-of-fit on F^2	1.195	1.130
Final R indexes [I>=2σ (I)]	R_1 = 0.0956, wR_2 = 0.2257	R_1 = 0.0595, wR_2 = 0.1528
Final R indexes [all data]	R_1 = 0.1022, wR_2 = 0.2292	R_1 = 0.0624, wR_2 = 0.1545
Largest diff. peak/hole / e Å^{-3}	2.13/-1.65	0.52/-0.32

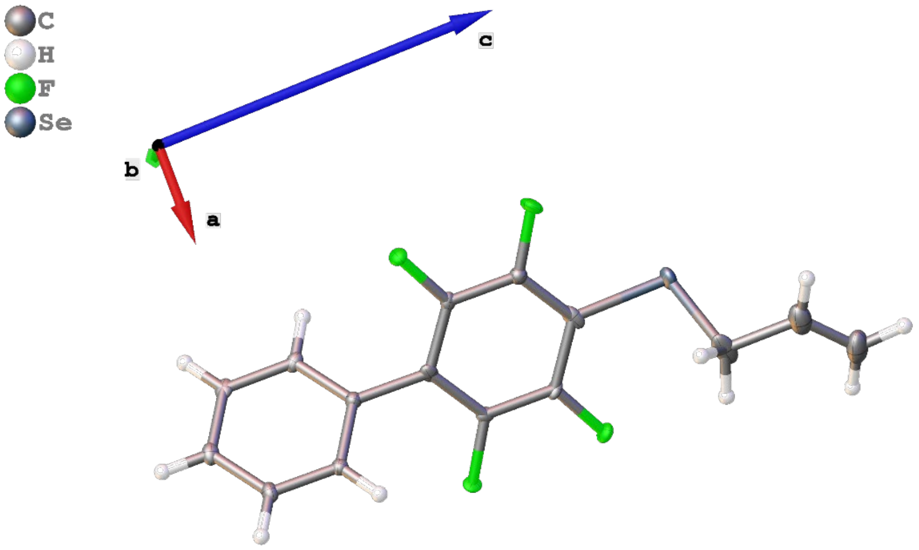

Figure S1. The structure of **Allyl-Se-Ph^{F}Ph**.

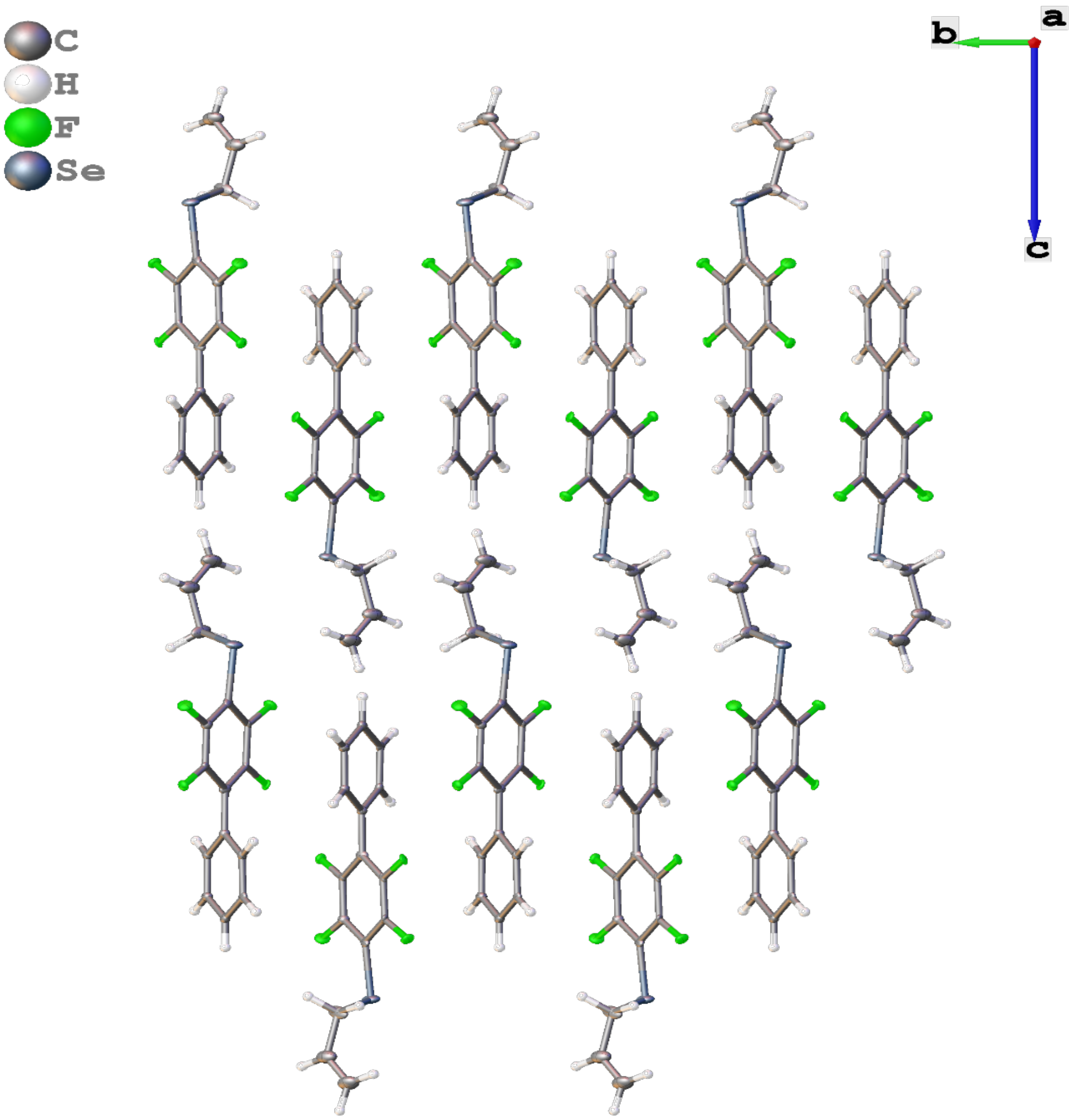

Figure S2. A fragment of a package in **Allyl-Se-Ph^{F}Ph** crystal.

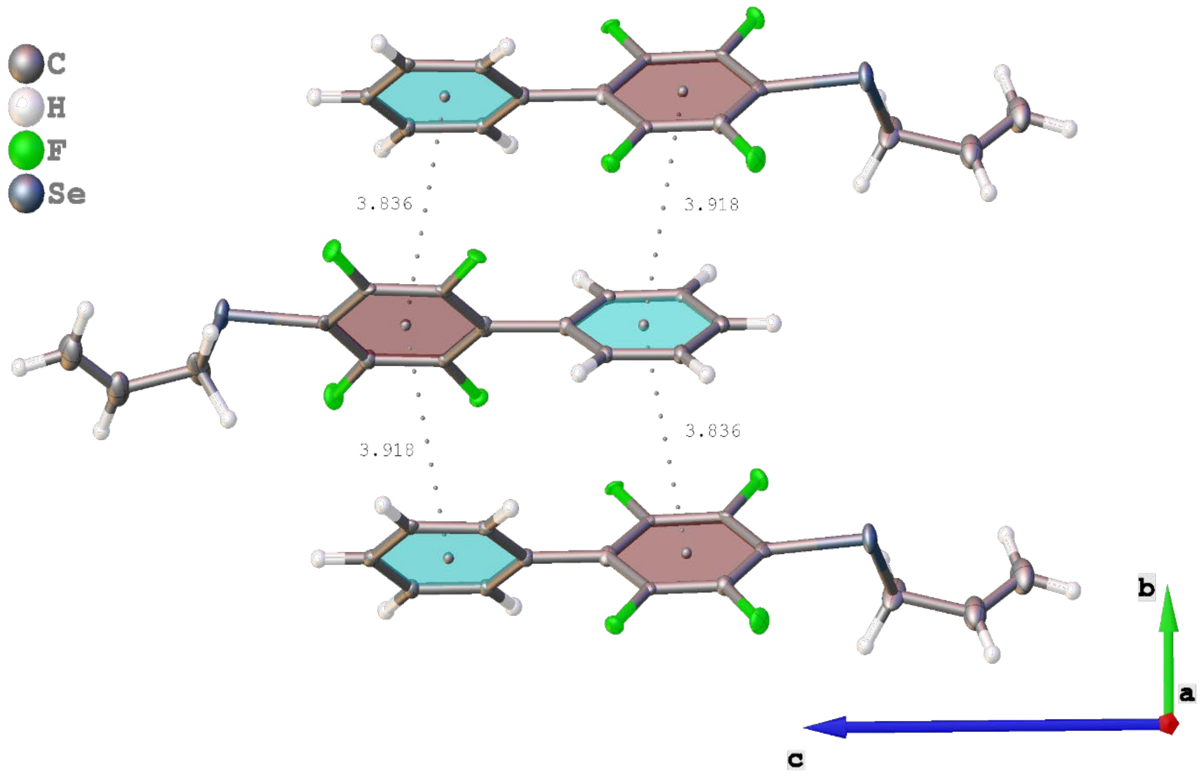

Figure S3. The aryl-perfluoroaryl interaction in **Allyl-Se-Ph^{F}Ph**.

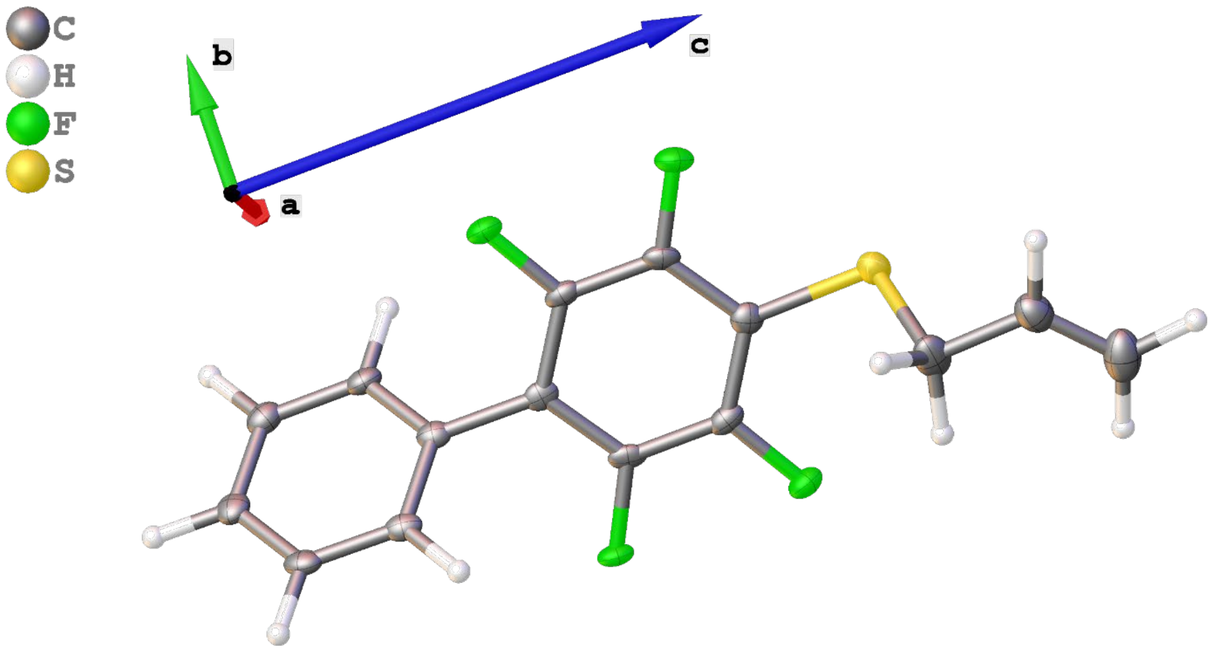

Figure S4. The structure of **Allyl-S-Ph^{F}Ph**.

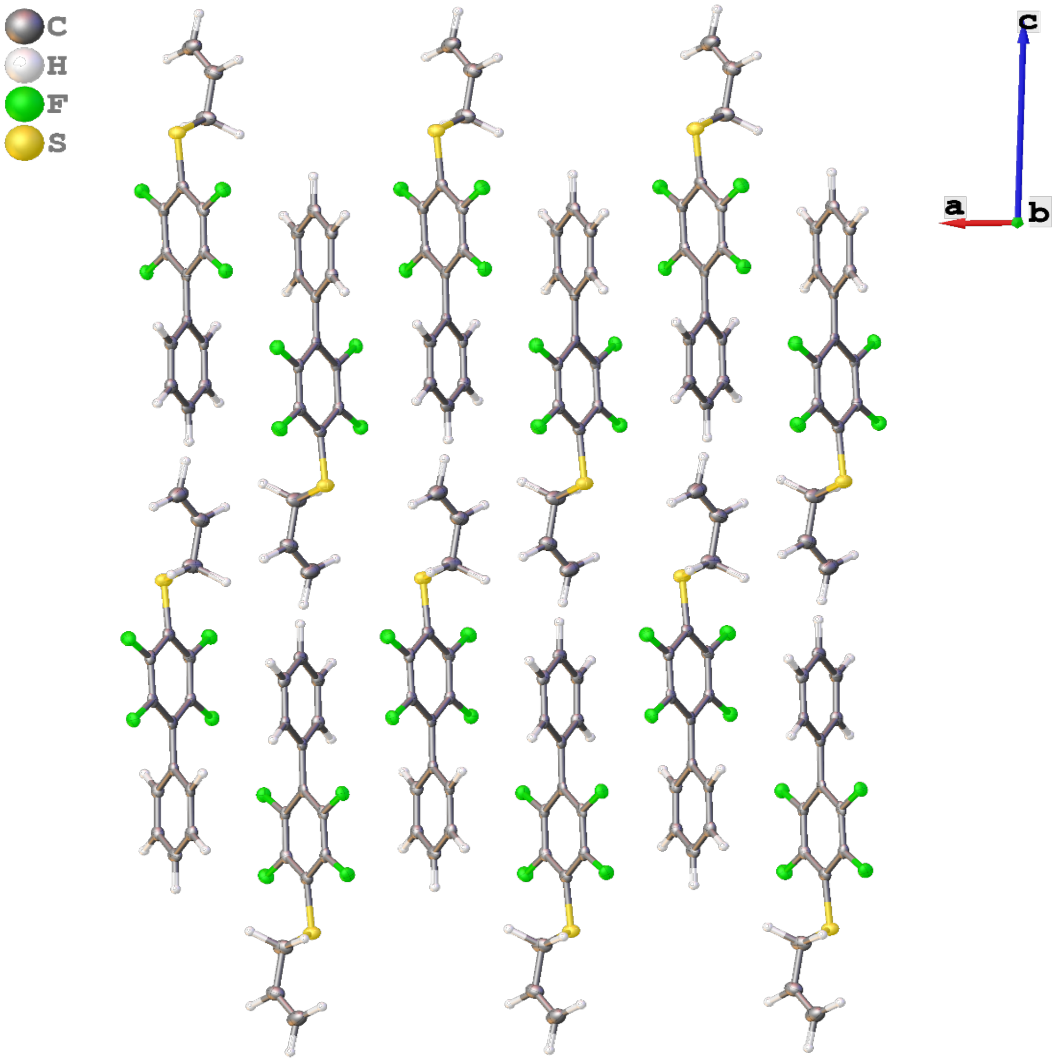

Figure S5. A fragment of a package in **Allyl-S-Ph^{F}Ph** crystal.

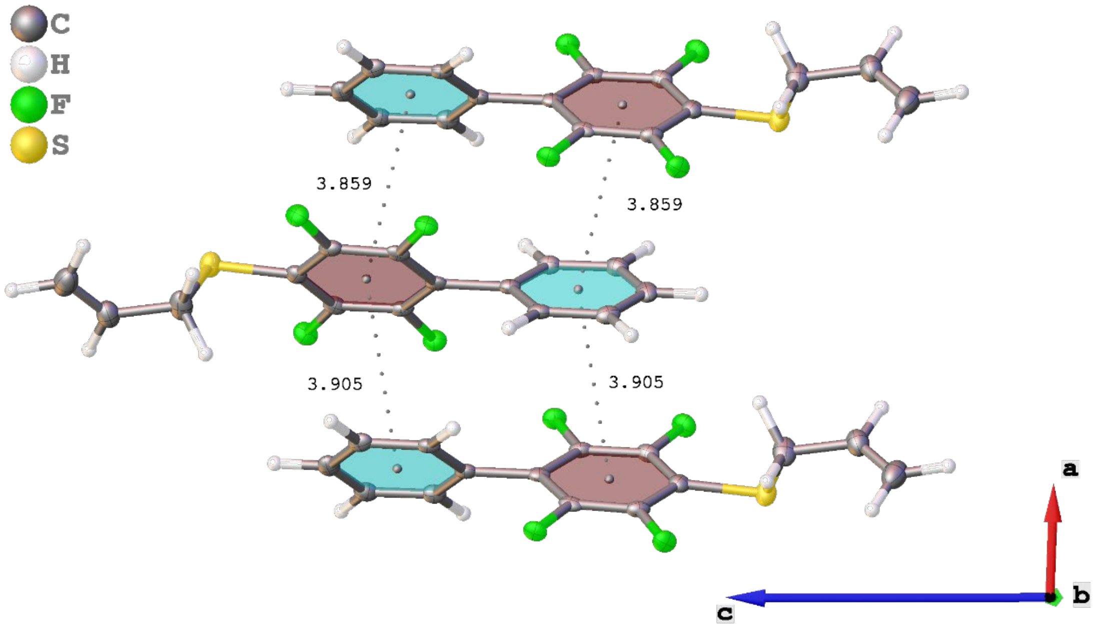

Figure S6. The aryl-perfluoroaryl interaction in **Allyl-S-Ph^{F}Ph**.

S2. NMR spectra

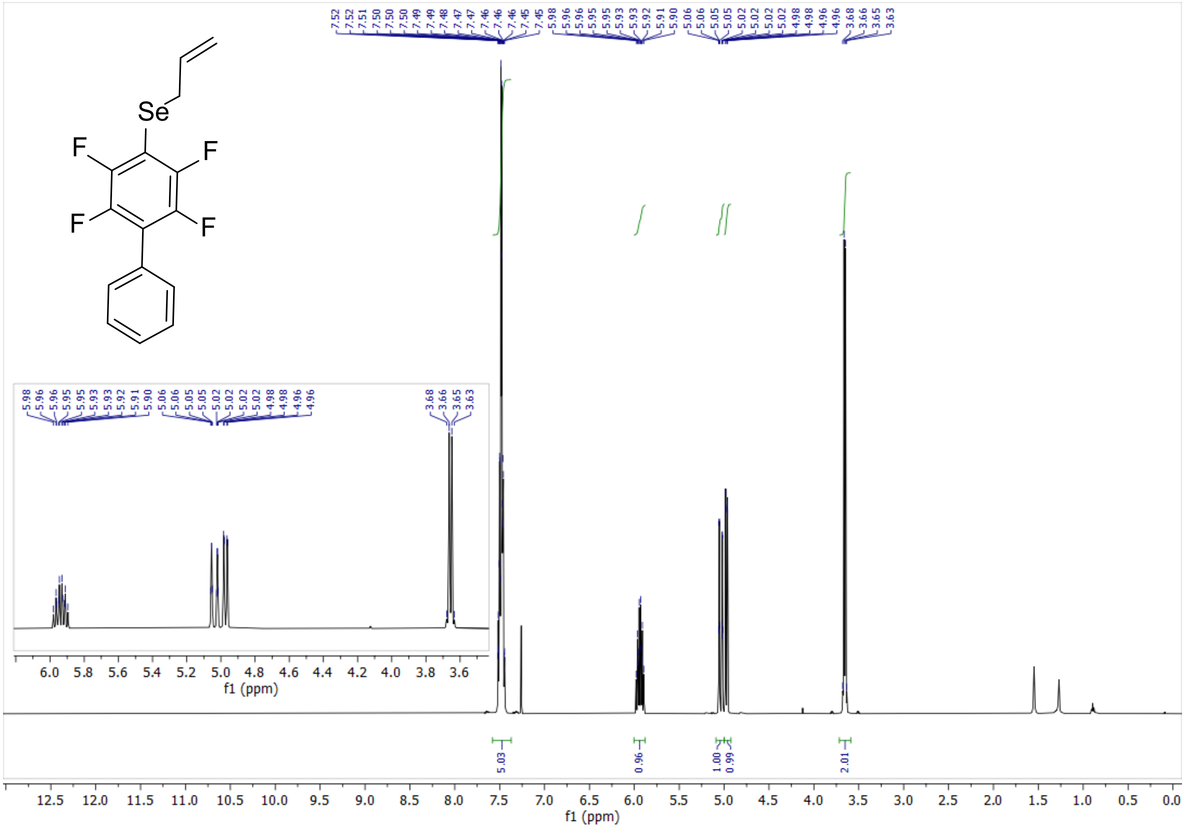

Figure S7. ^{1}H NMR (400 MHz, $CDCl_3$) spectrum of **Allyl-Se-Ph^{F}Ph**.

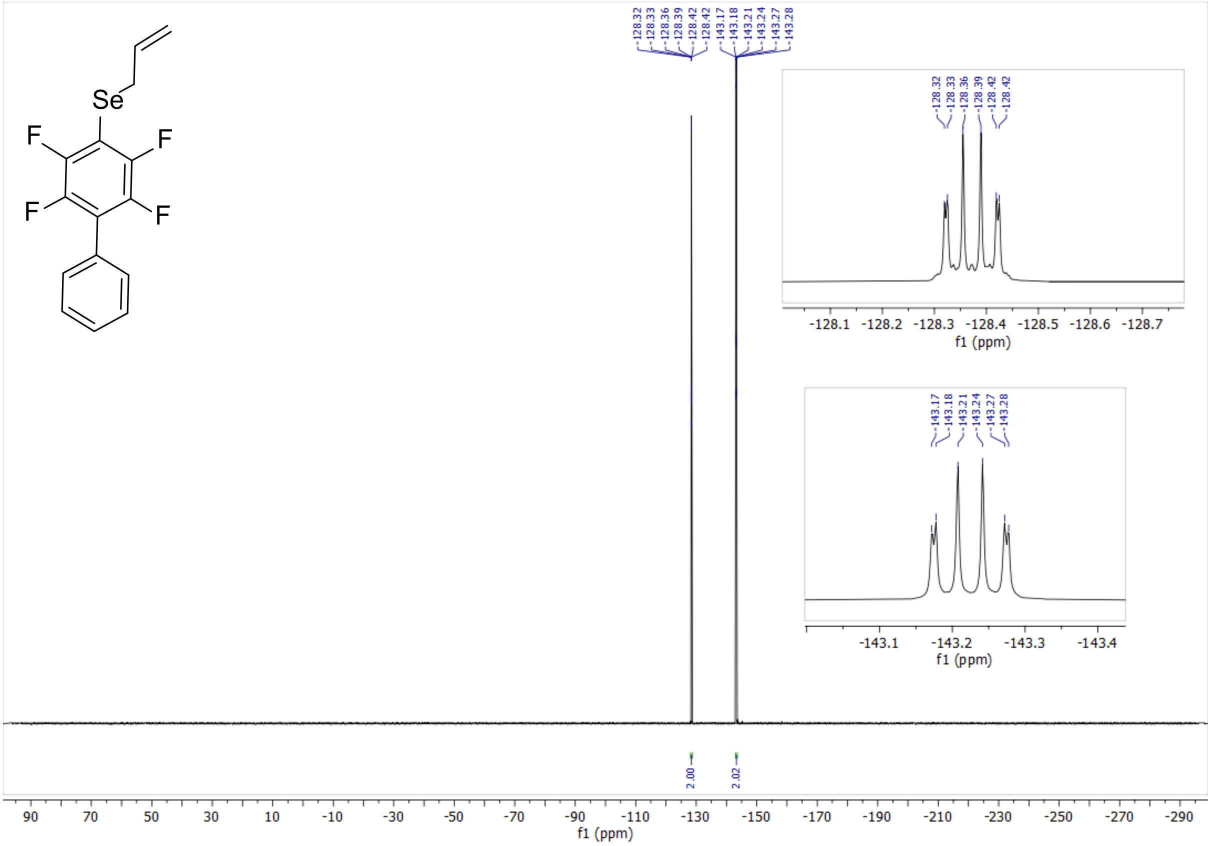

Figure S8. $^{19}F\{^{1}H\}$ NMR (376 MHz, $CDCl_3$) spectrum of **Allyl-Se-Ph^{F}Ph**.

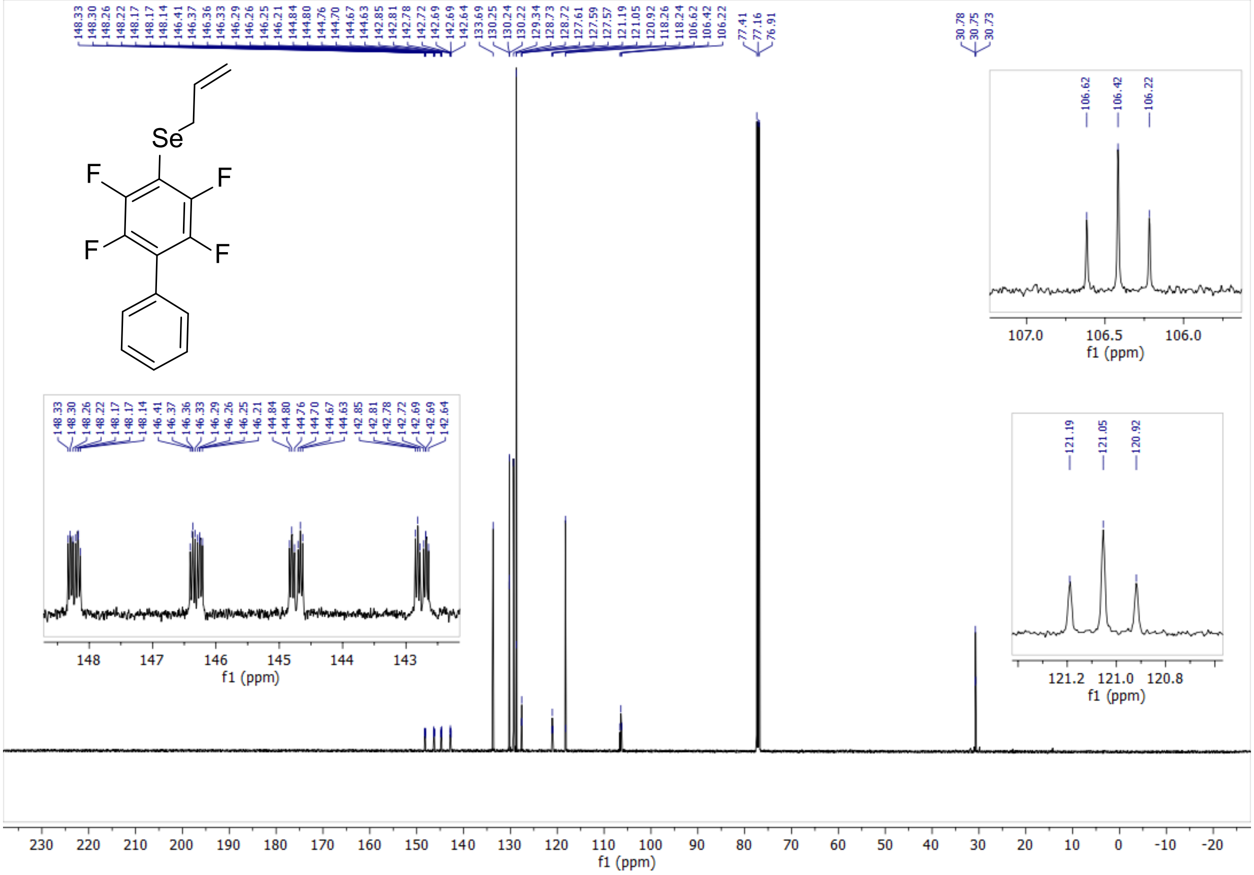

Figure S9. $^{13}C\{^{1}H\}$ NMR (126 MHz, $CDCl_3$) spectrum of **Allyl-Se-Ph^{F}Ph**.

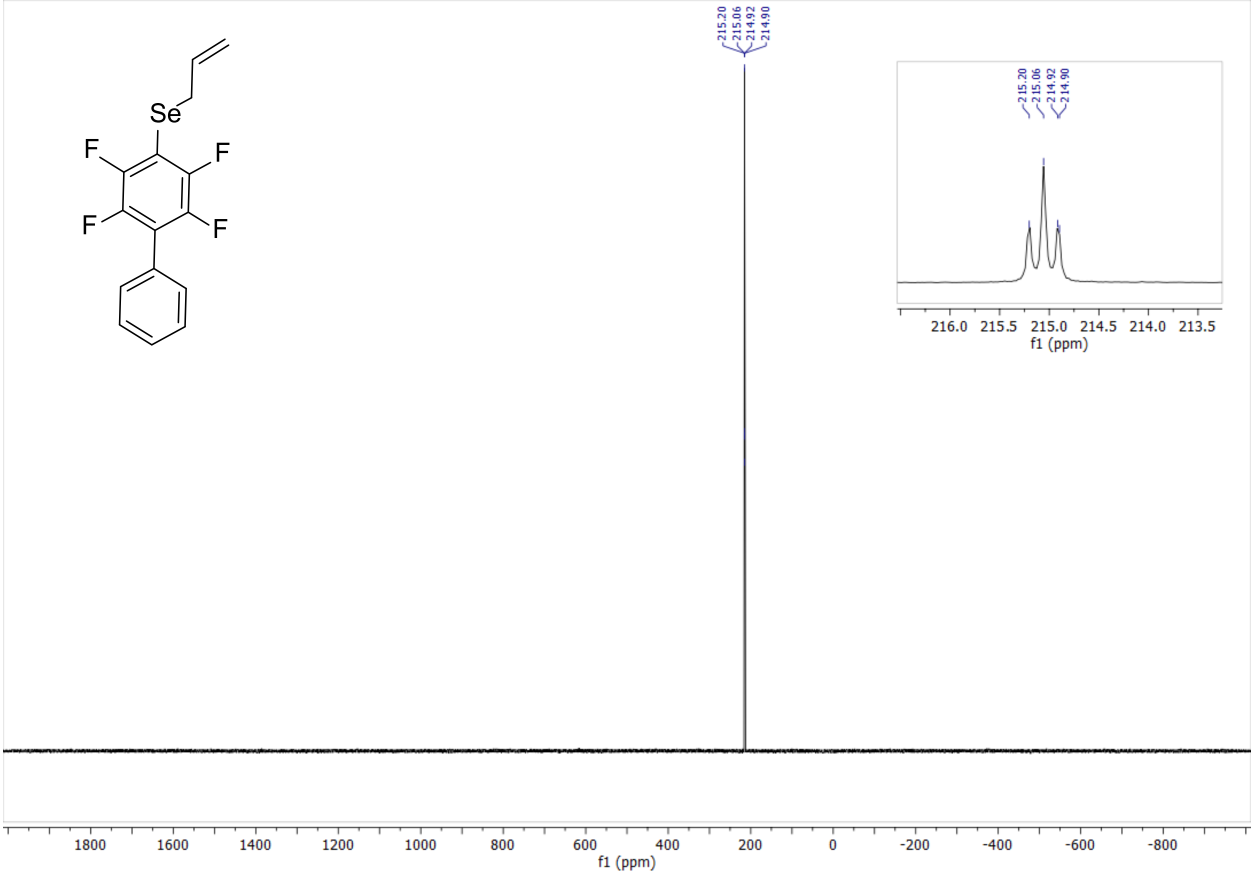

Figure S10. ^{77}Se NMR (95 MHz, $CDCl_3$) spectrum of **Allyl-Se-Ph^{F}Ph**.

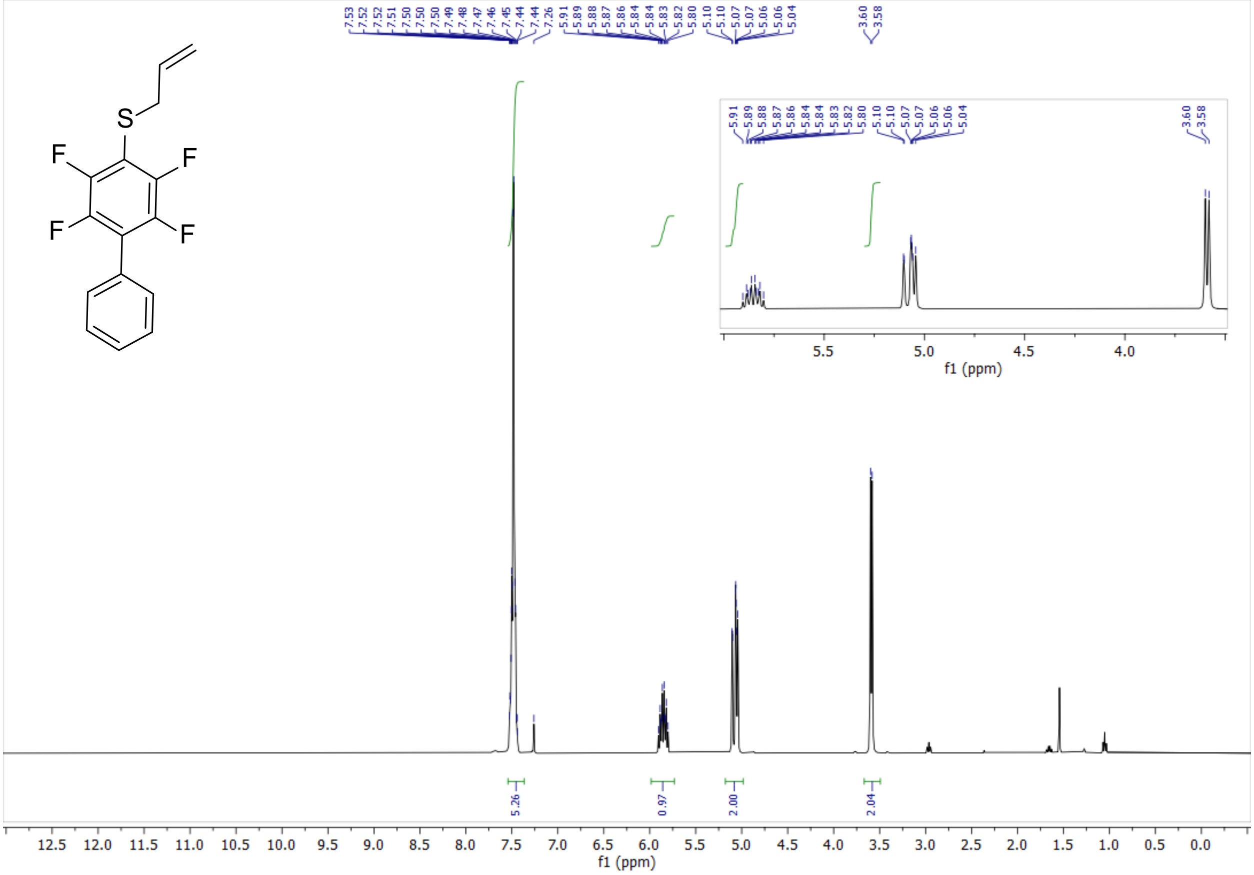

Figure S11. ^{1}H NMR (400 MHz, $CDCl_3$) spectrum of **Allyl-S-Ph^{F}Ph**.

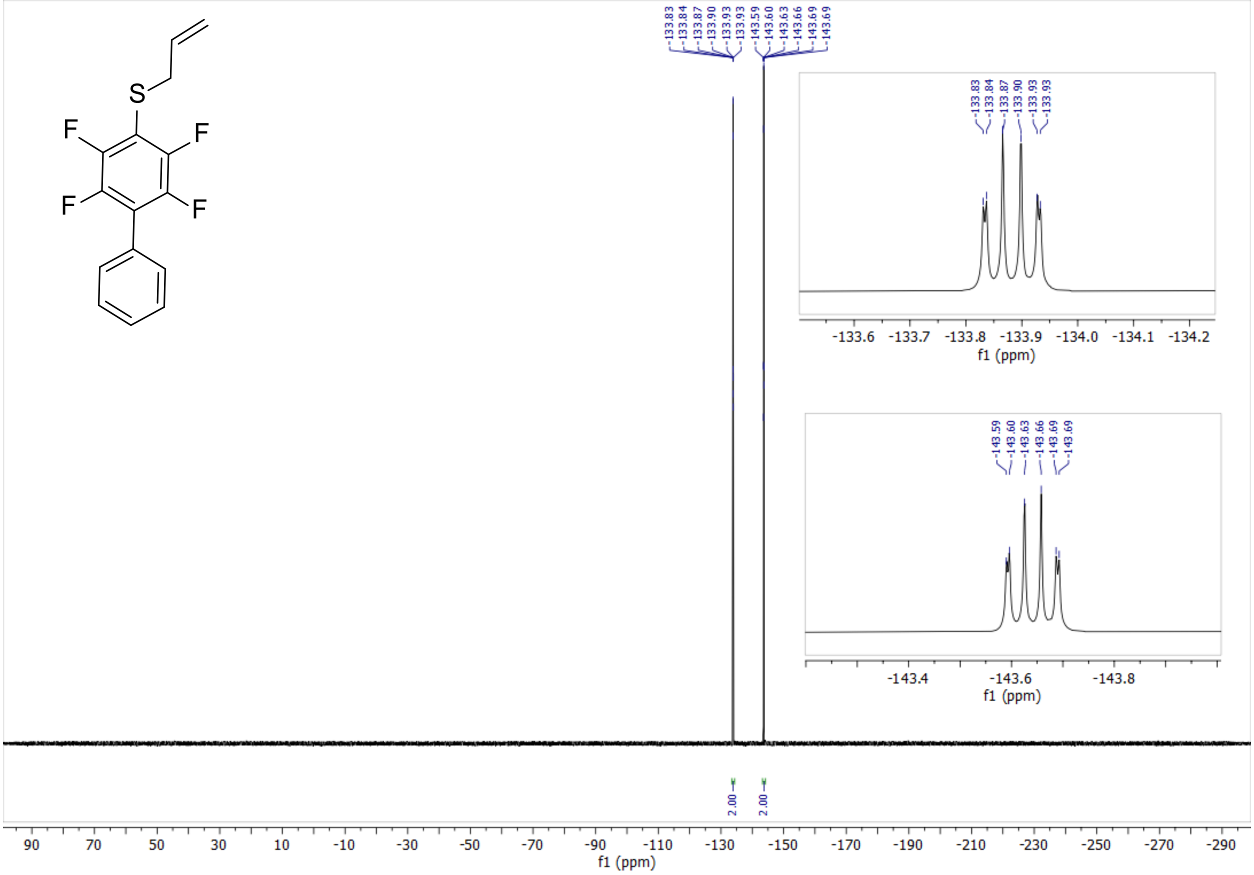

Figure S12. ^{19}F{^{1}H} NMR (376 MHz, $CDCl_3$) spectrum of **Allyl-S-Ph^{F}Ph**.

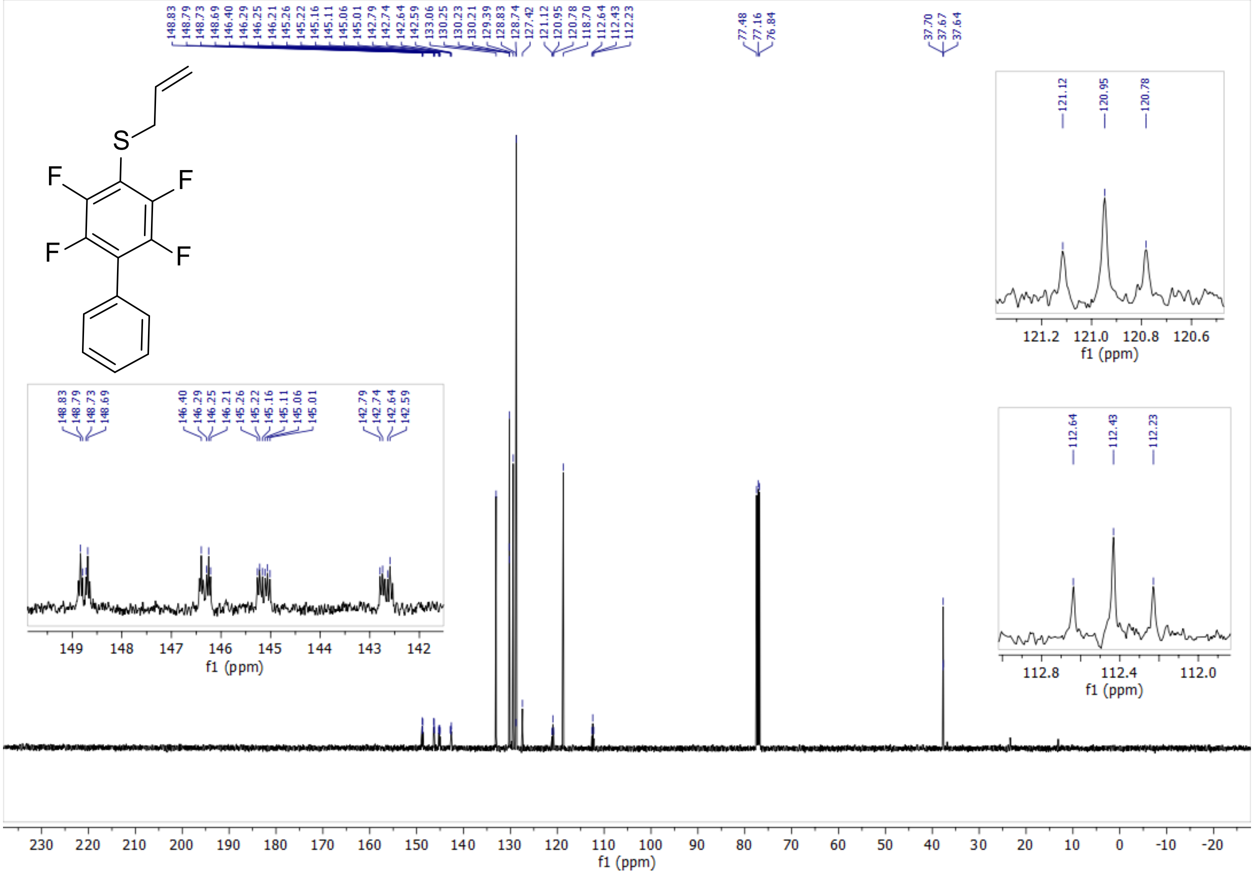

Figure S13. $^{13}C\{^{1}H\}$ NMR (101 MHz, $CDCl_3$) spectrum of **Allyl-S-Ph^FPh**.

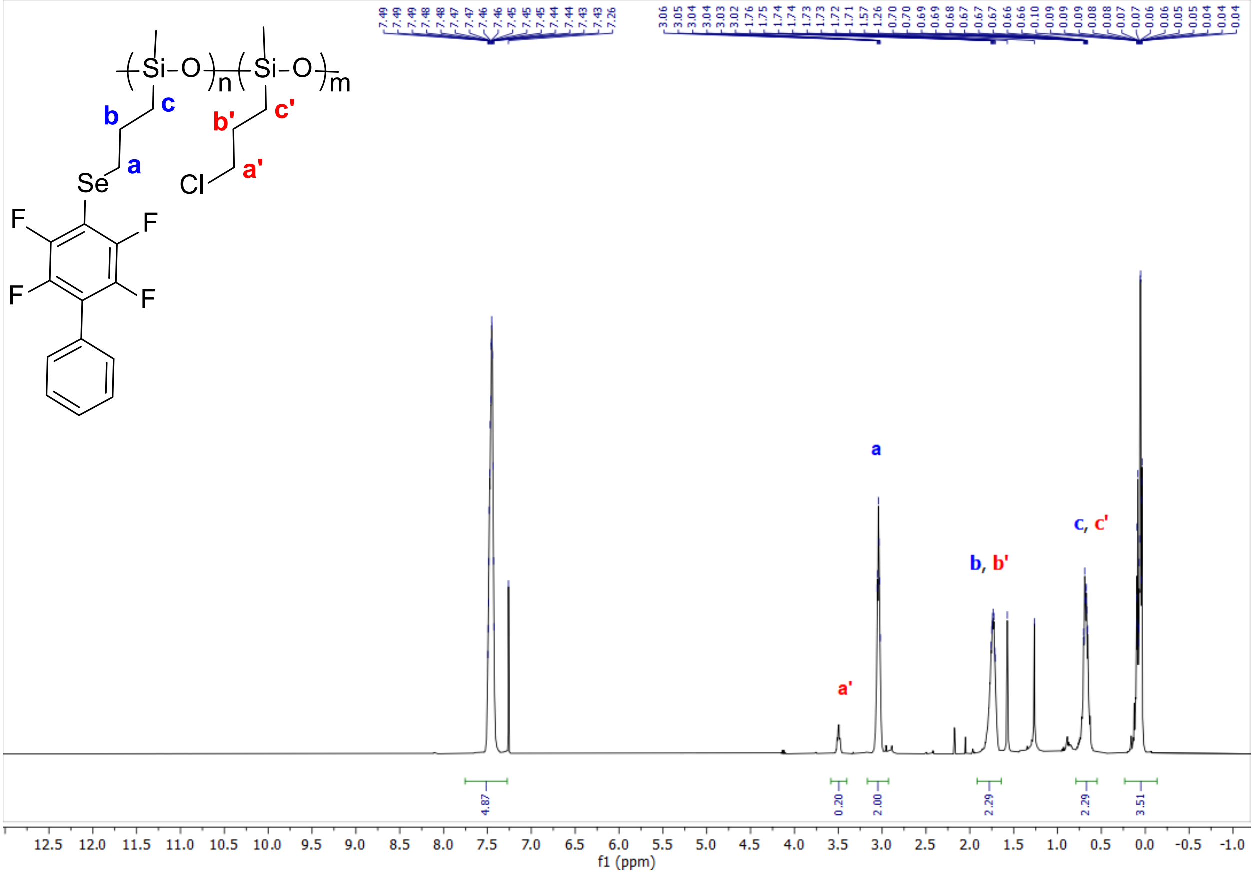

Figure S14. ^{1}H NMR (500 MHz, $CDCl_3$) spectrum of **Ph^FPh-Se-PMS**.

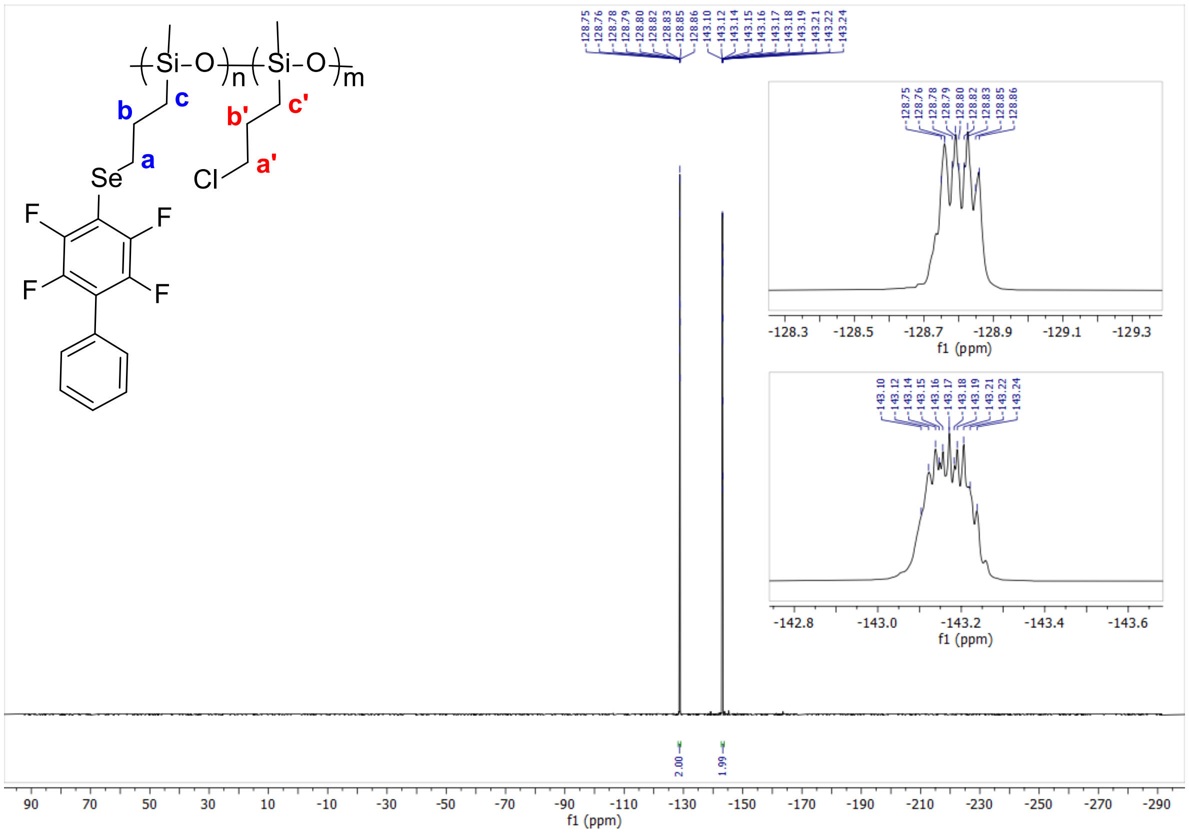

Figure S15. $^{19}F\{^{1}H\}$ NMR (376 MHz, $CDCl_3$) spectrum of **Ph^{F}Ph-Se-PMS**.

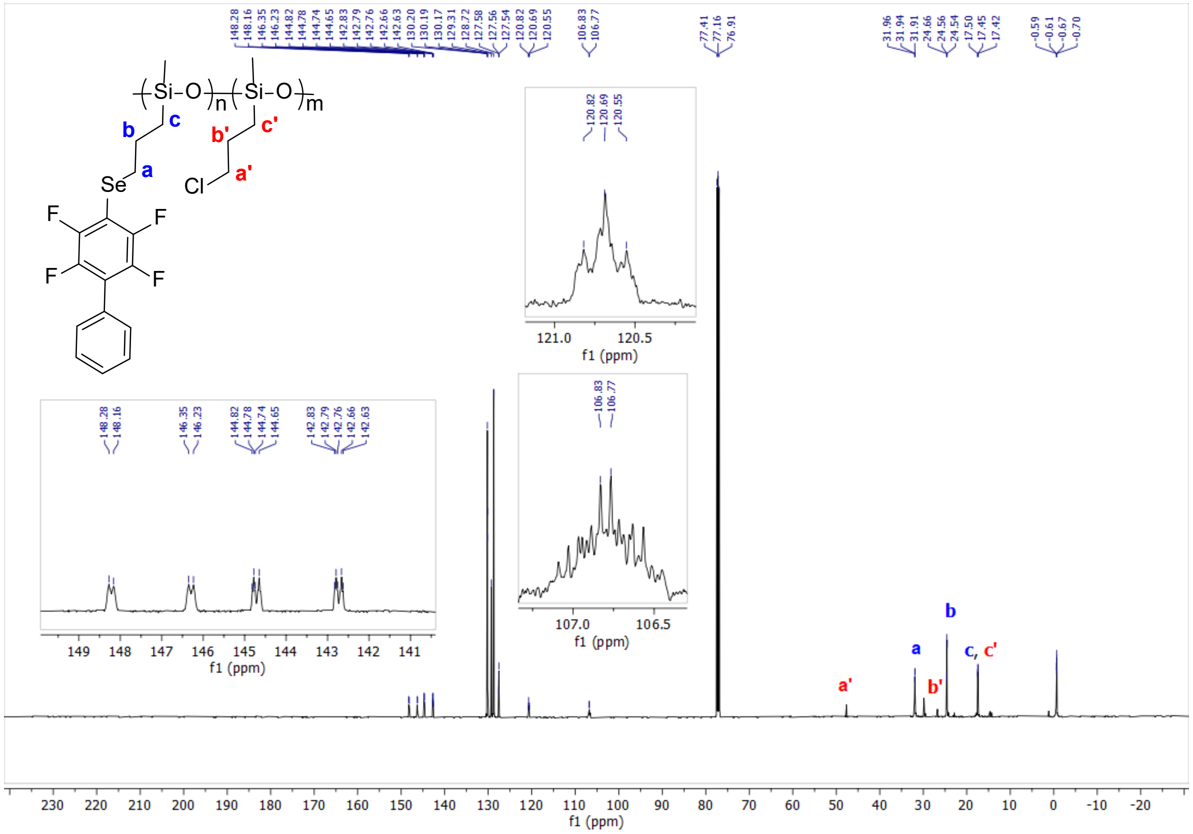

Figure S16. $^{13}C\{^{1}H\}$ NMR (126 MHz, $CDCl_3$) spectrum of **Ph^{F}Ph-Se-PMS**.

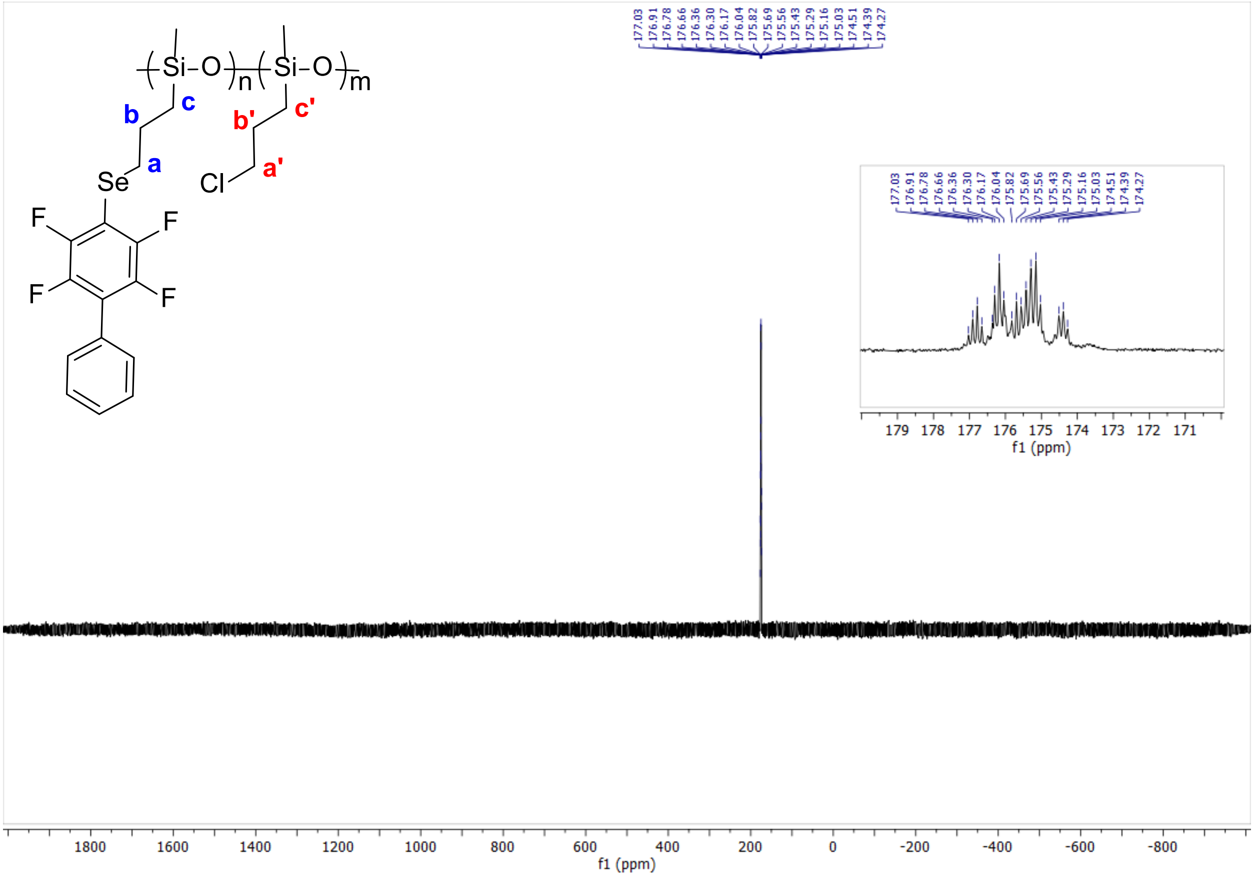

Figure S17. ^{77}Se NMR (95 MHz, $CDCl_3$) spectrum of **Ph^{F}Ph-Se-PMS**.

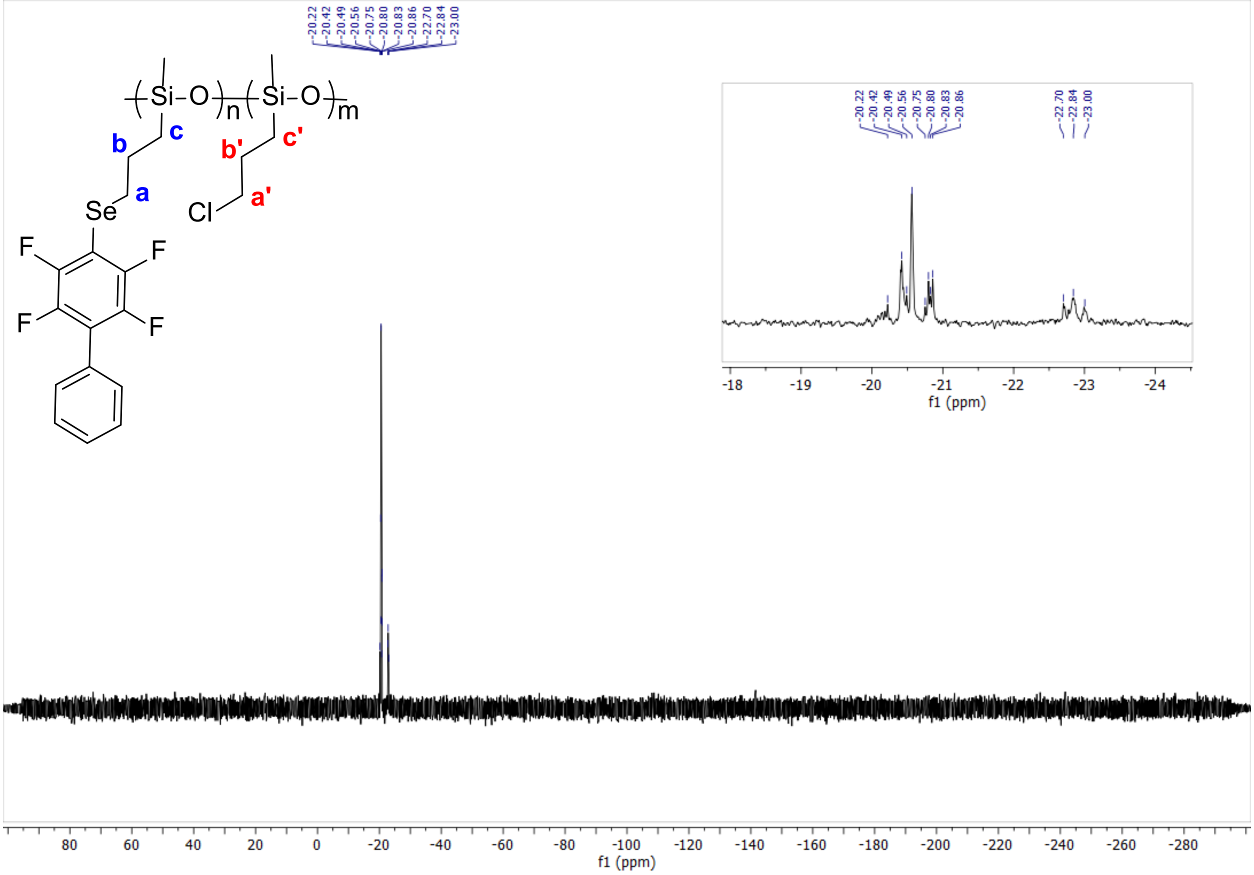

Figure S18. ^{29}Si NMR (79 MHz, $CDCl_3$) spectrum of **Ph^{F}Ph-Se-PMS**.

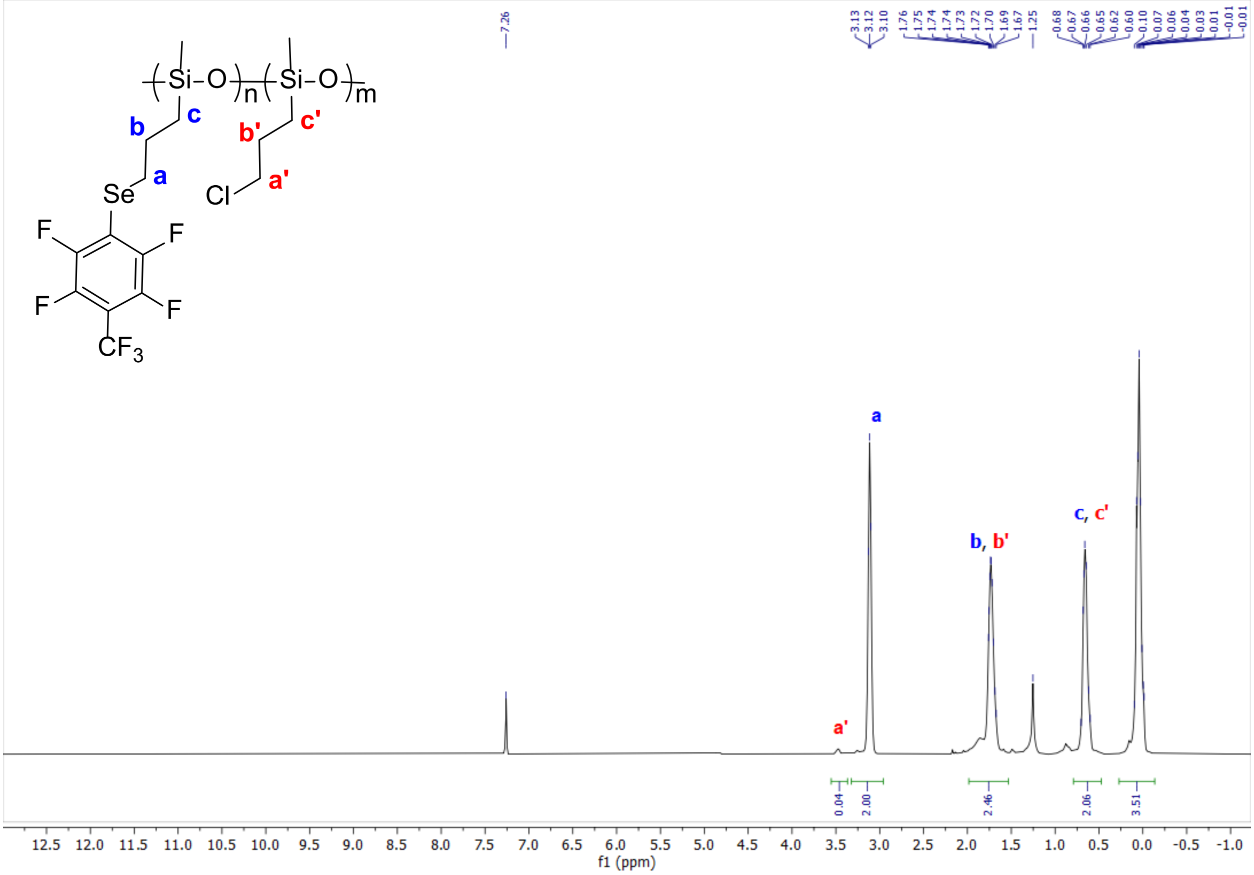

Figure S19. ^{1}H NMR (500 MHz, $CDCl_3$) spectrum of **CF_3Ph^F-Se-PMS**.

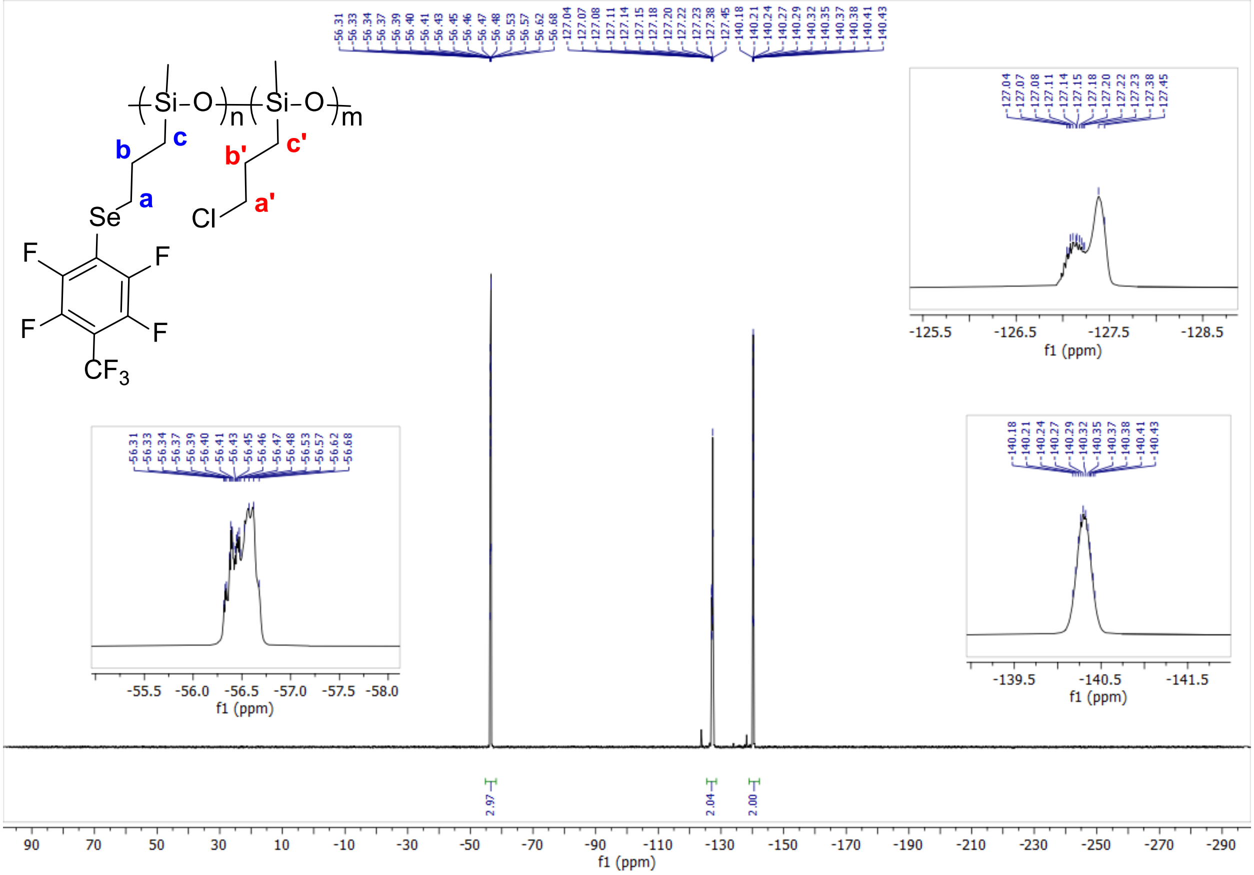

Figure S20. $^{19}F\{^1H\}$ NMR (376 MHz, $CDCl_3$) spectrum of **CF_3Ph^F-Se-PMS**.

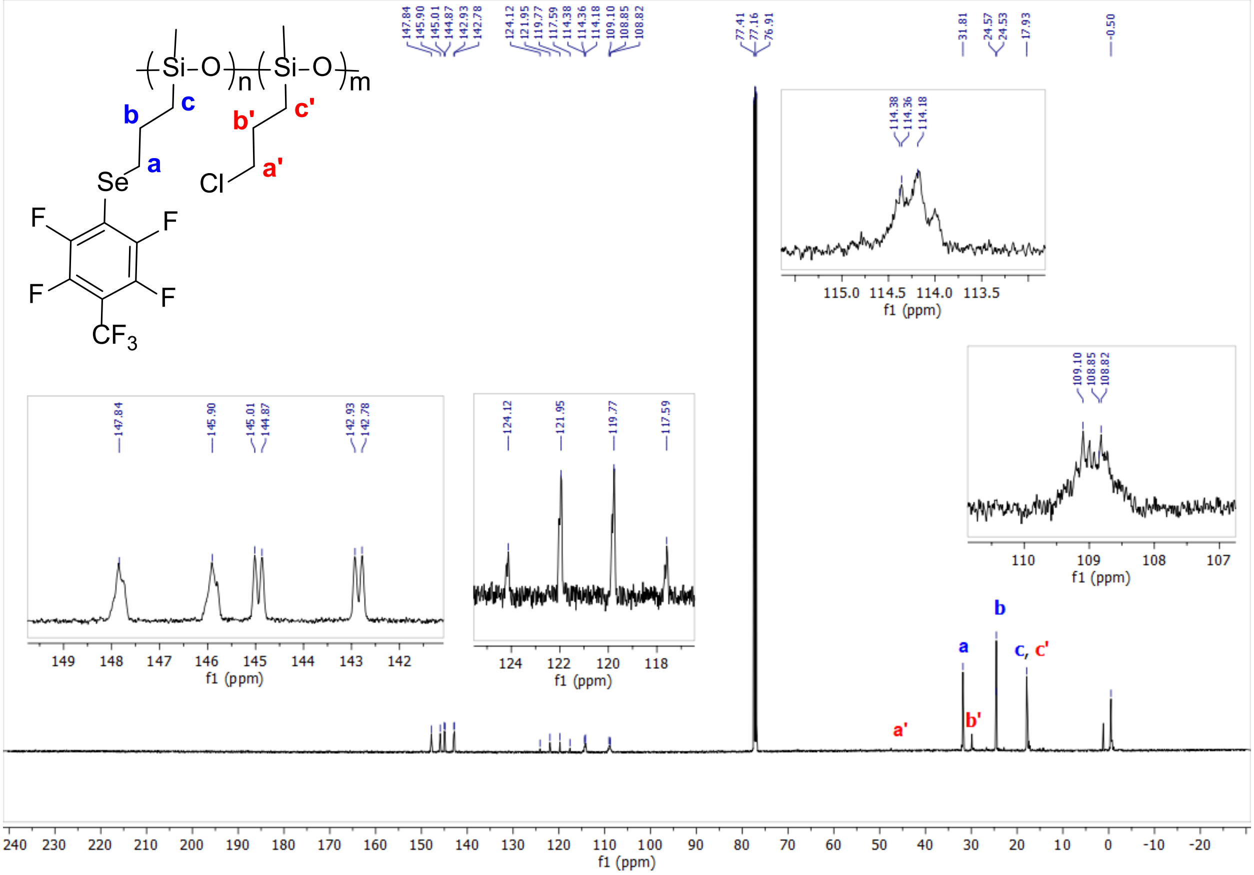

Figure S21. $^{13}C\{^1H\}$ NMR (126 MHz, $CDCl_3$) spectrum of **CF_3Ph^F-Se-PMS**.

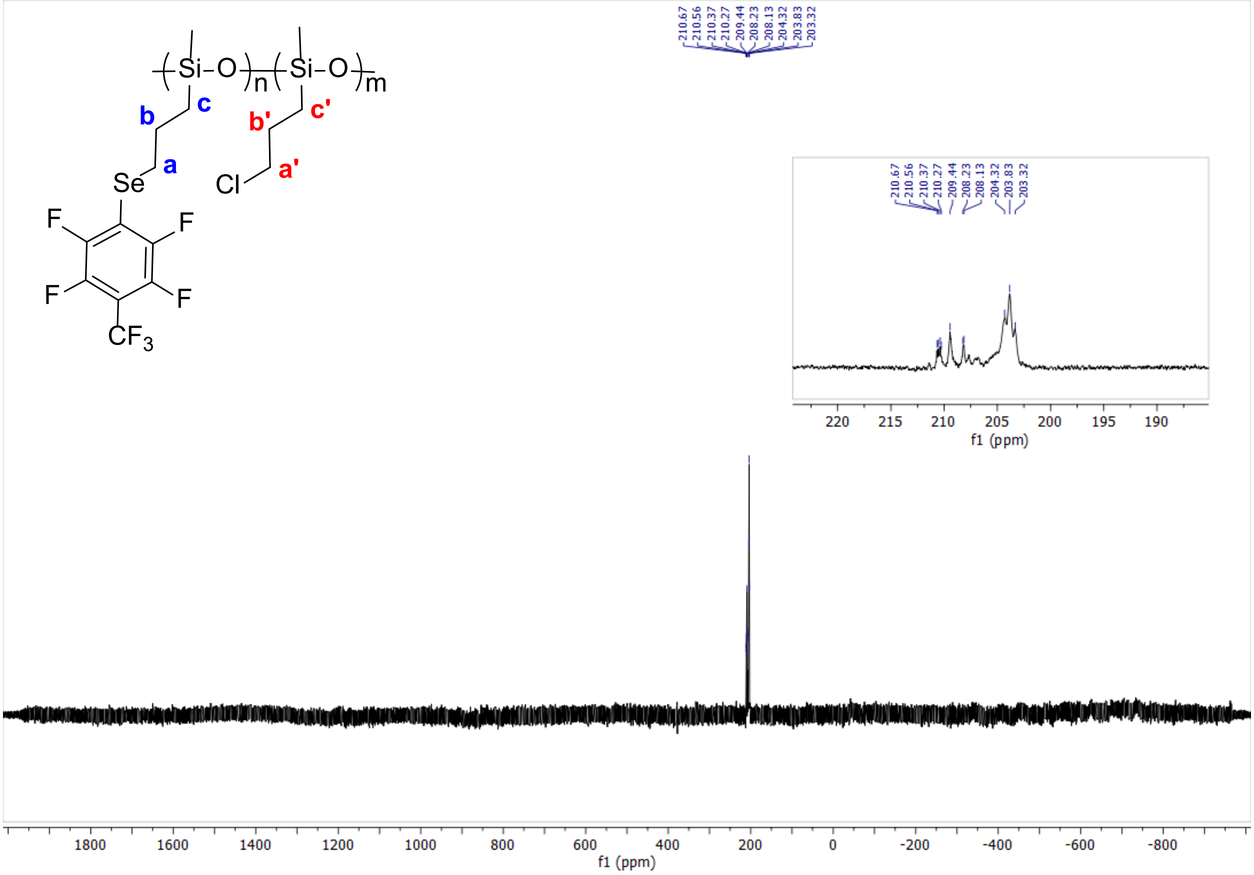

Figure S22. ^{77}Se NMR (95 MHz, $CDCl_3$) spectrum of **CF_3Ph^F-Se-PMS**.

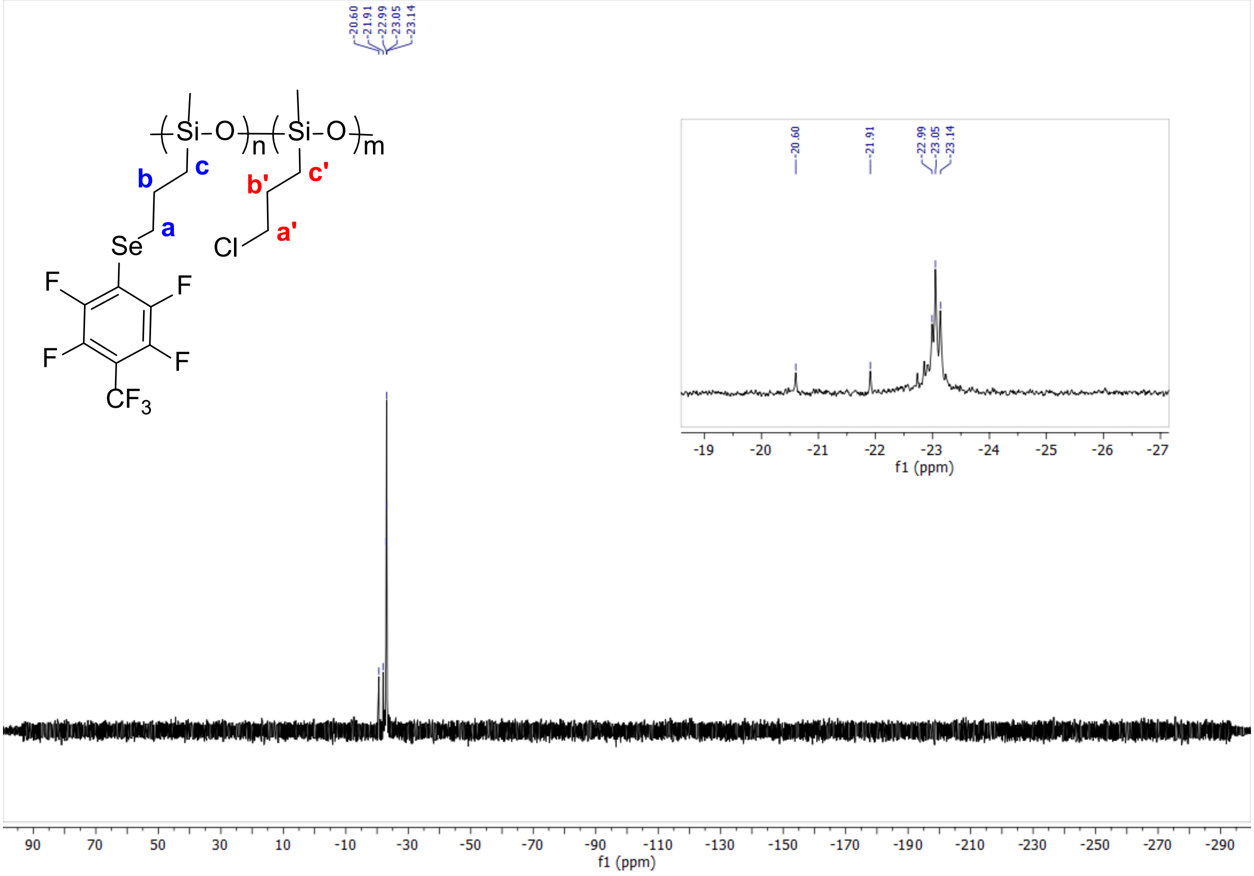

Figure S23. ^{29}Si NMR (99 MHz, $CDCl_3$) spectrum of **CF_3Ph^F-Se-PMS**.

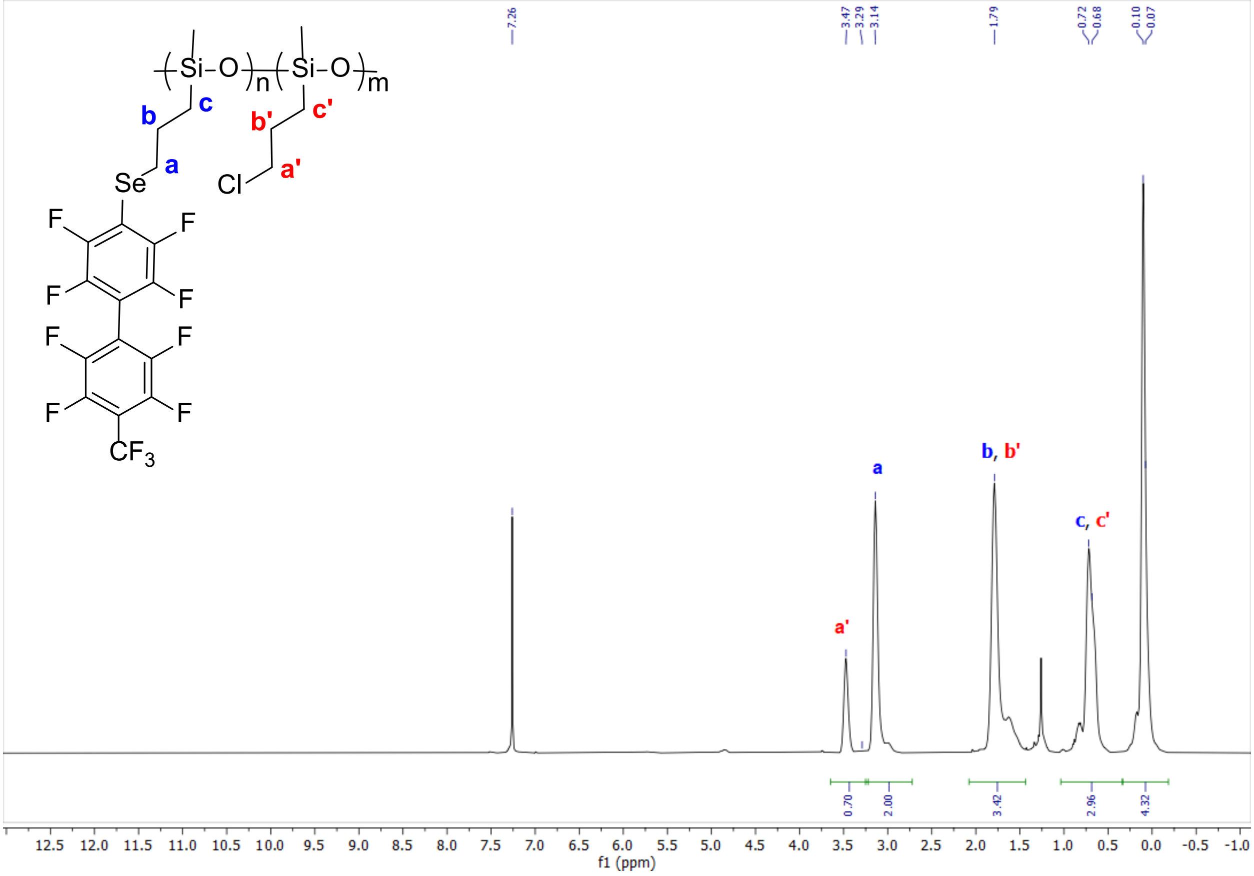

Figure S24. ^{1}H NMR (400 MHz, $CDCl_3$) spectrum of **$CF_3Ph^FPh^F$-Se-PMS**.

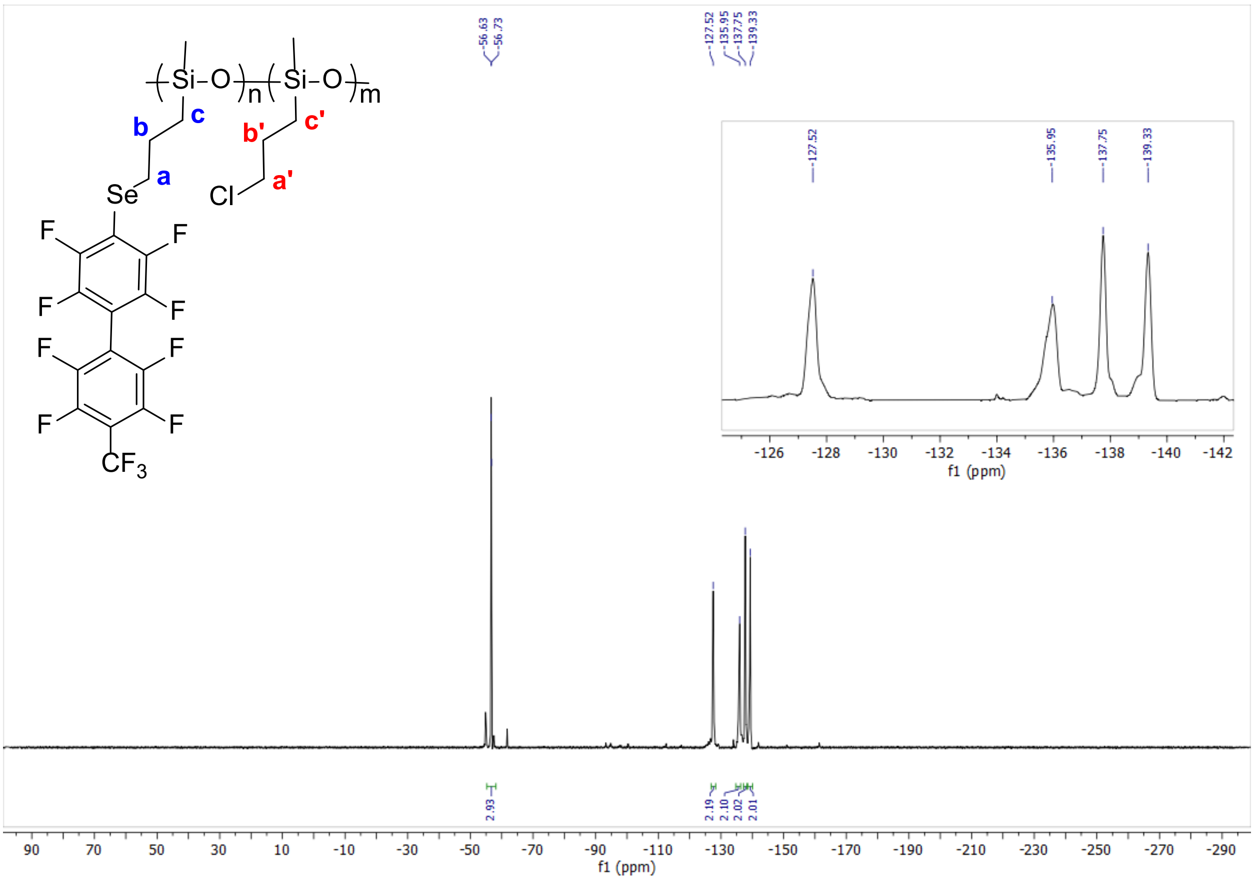

Figure S25. $^{19}F\{^1H\}$ NMR (376 MHz, $CDCl_3$) spectrum of **$CF_3Ph^FPh^F$-Se-PMS**.

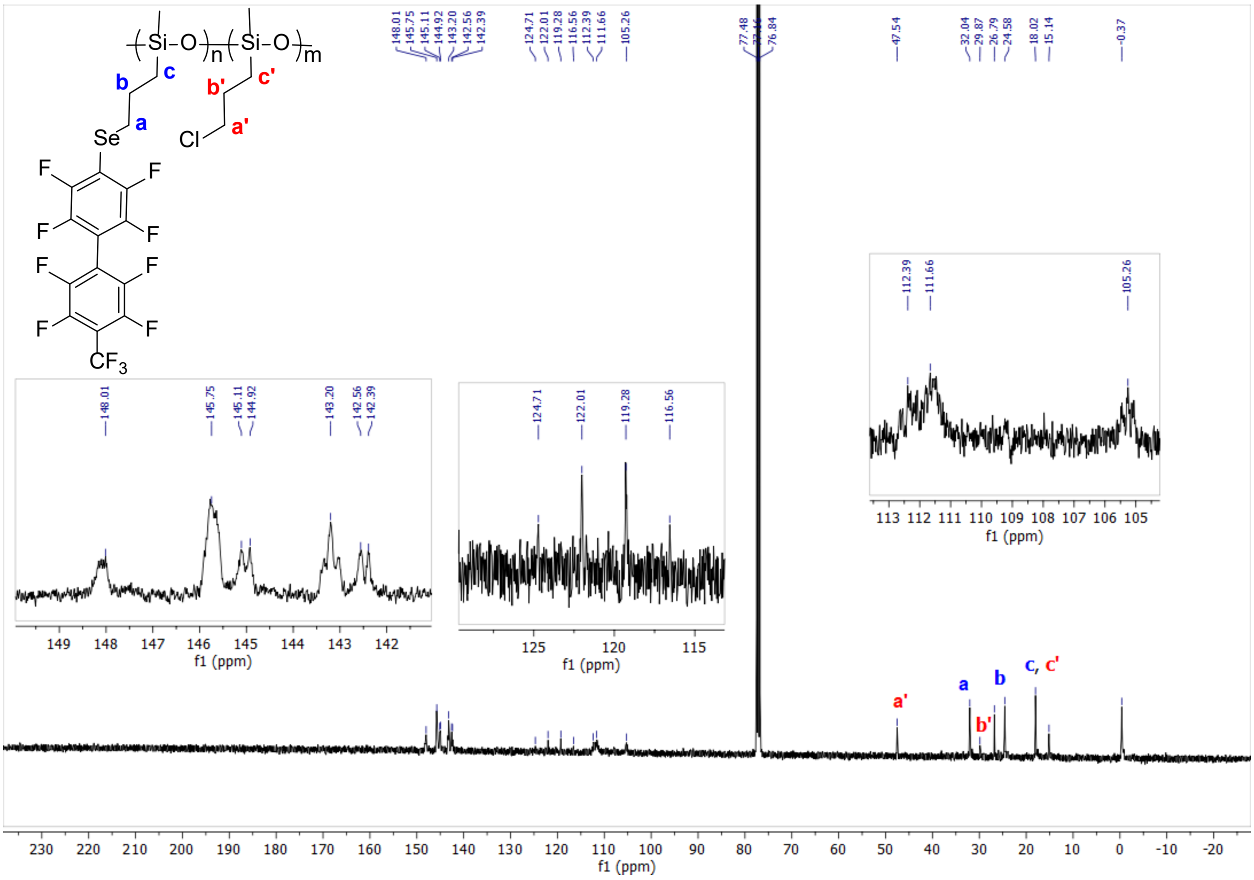

Figure S26. $^{13}C\{^1H\}$ NMR (101 MHz, $CDCl_3$) spectrum of **$CF_3Ph^FPh^F$-Se-PMS**.

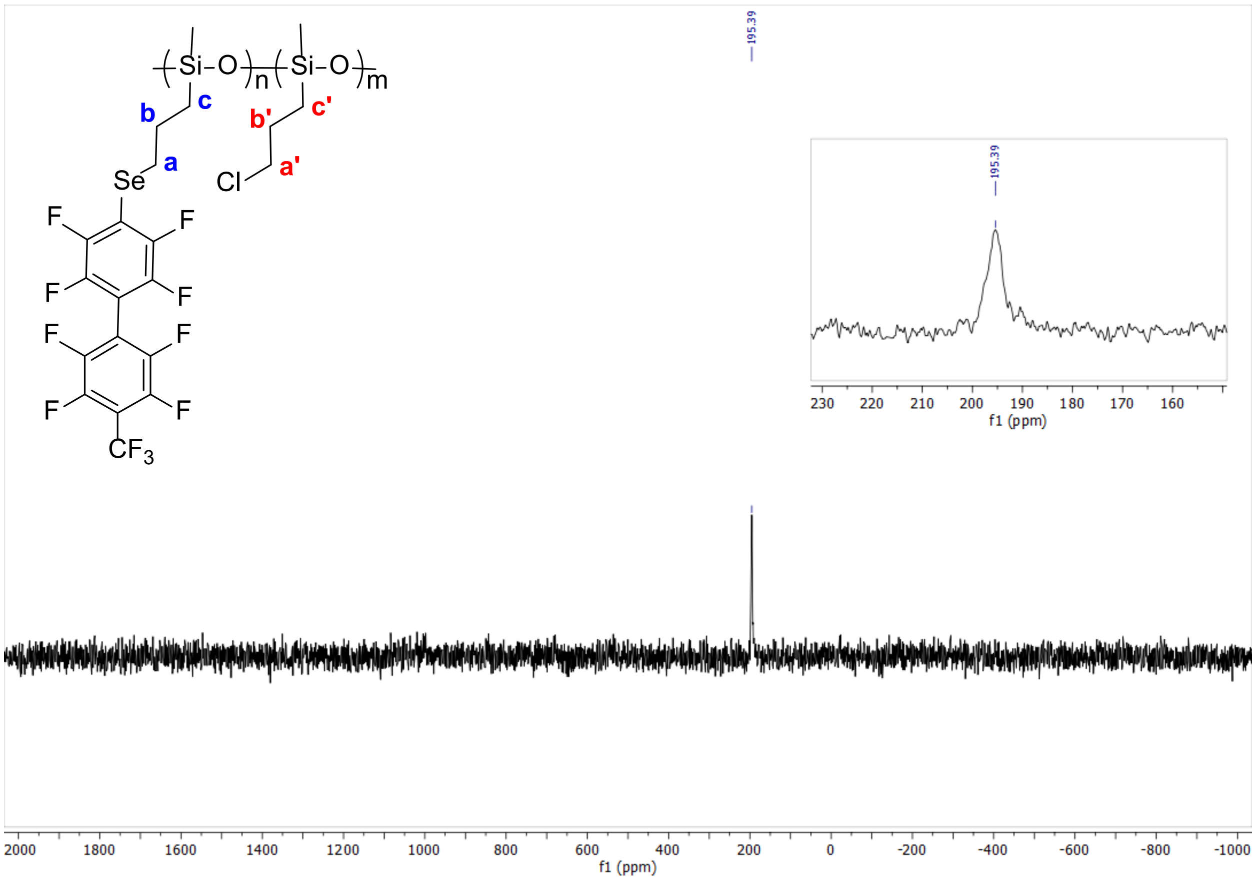

Figure S27. ^{77}Se NMR (76 MHz, $CDCl_3$) spectrum of **$CF_3Ph^FPh^F$-Se-PMS**.

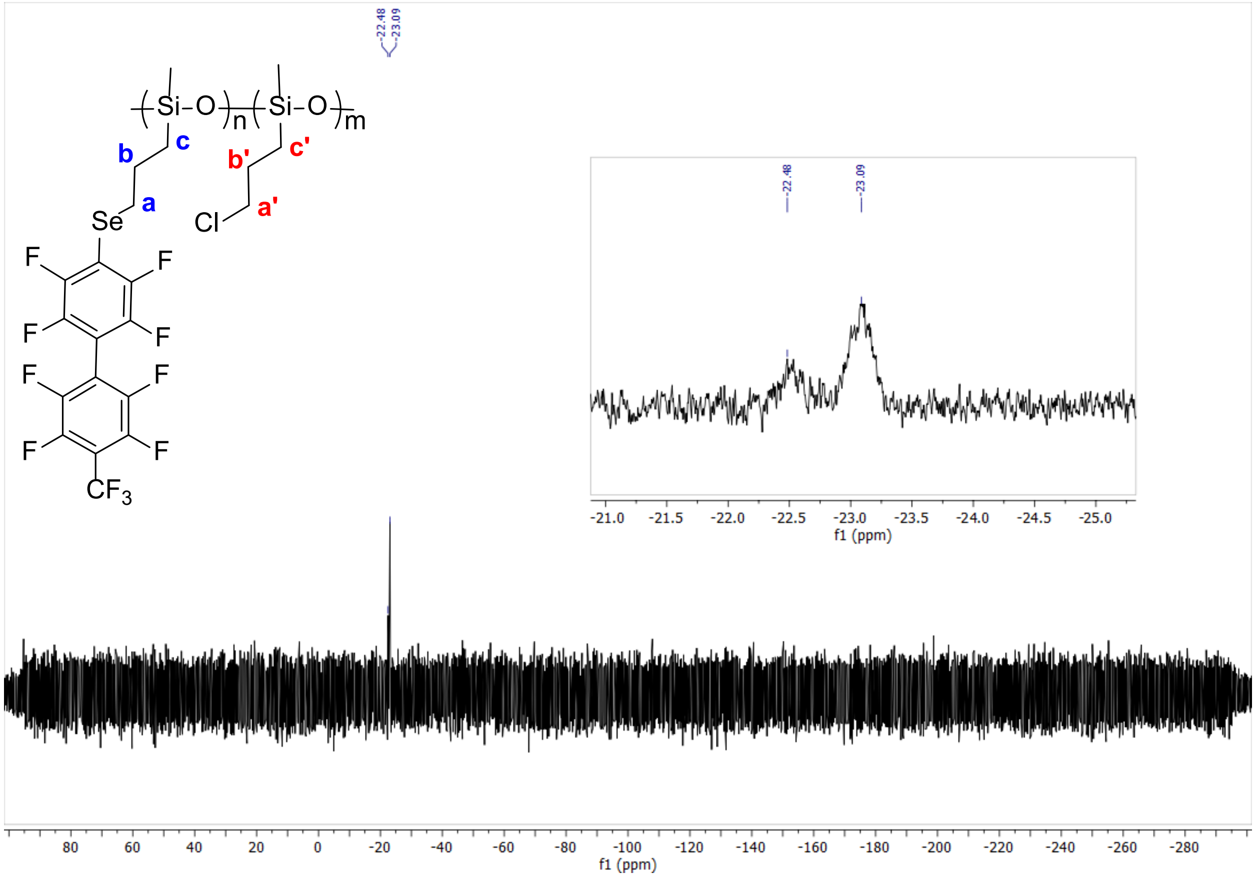

Figure S28. ^{29}Si NMR (79 MHz, $CDCl_3$) spectrum of **$CF_3Ph^FPh^F$-Se-PMS**.

S3. Molecular weights of polymers

Table S2. Number-average and weight-average molecular weights and dispersity (*Đ*) of the polymers

Polymer	Area, %	M_n	M_w	*Đ*
**Cl-PMS**	87	6600	8730	1.32
	13	540	650	1.22
**CF_3Ph^F-Se-PMS**	66	10040	15560	1.55
	29	1650	1810	1.10
	5	350	420	1.21
**$CF_3Ph^FPh^F$-Se-PMS**	75	10420	14440	1.39
	25	500	680	1.38
**$PhPh^F$-Se-PMS**	97	1140	1240	1.09

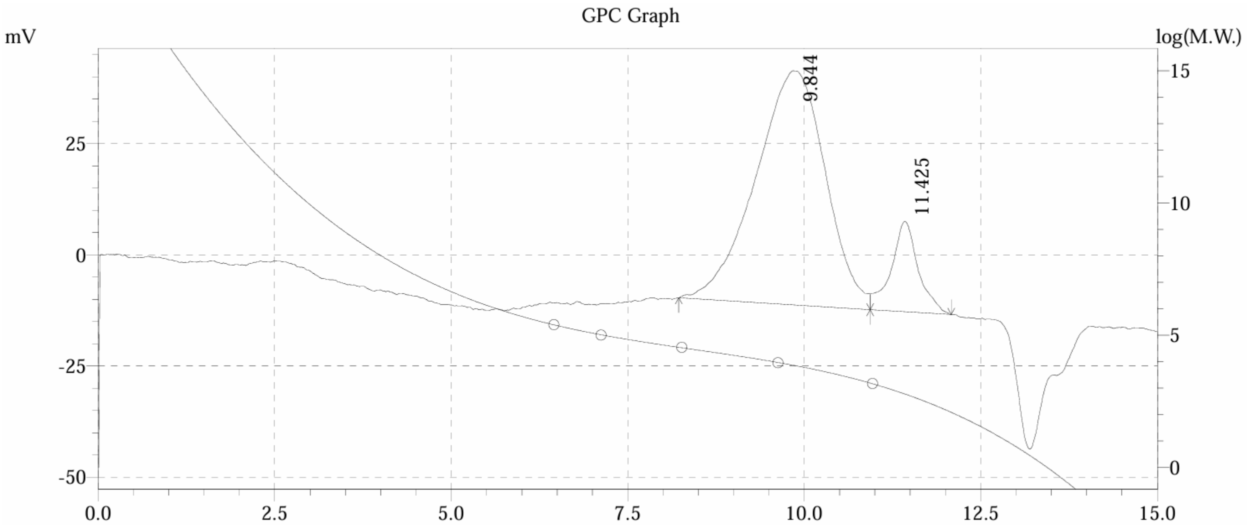

Figure S29. GPC graphic of **Cl-PMS**.

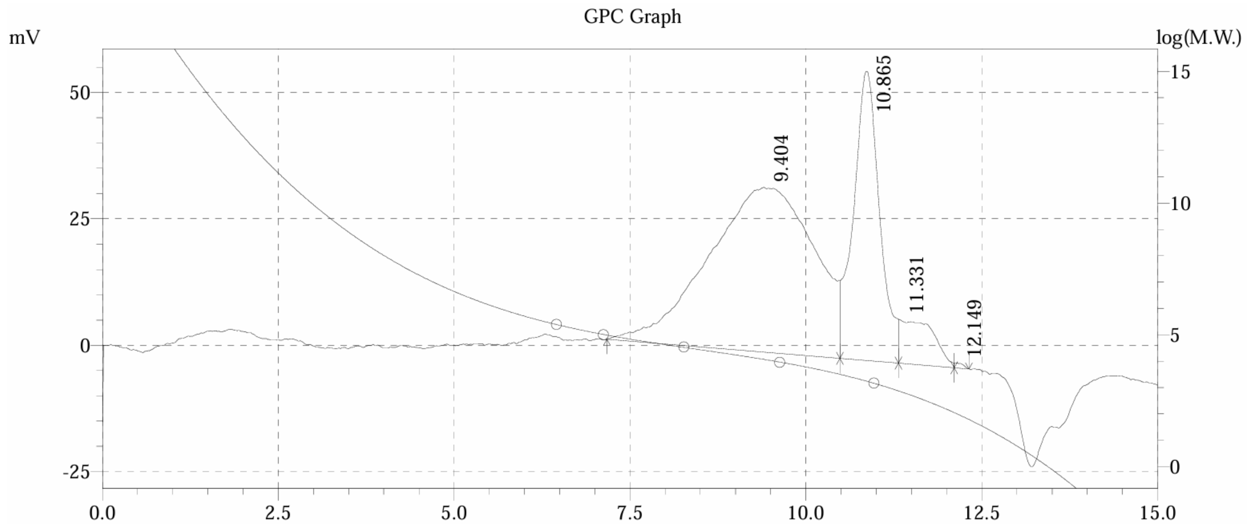

Figure S30. GPC graphic of **CF_3Ph^F-Se-PMS**.

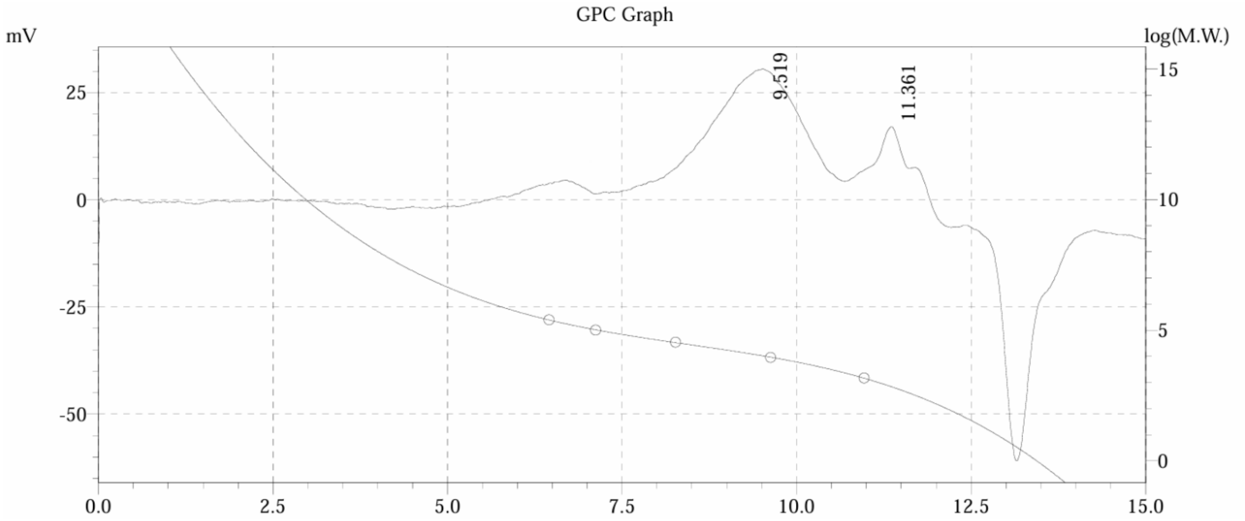

Figure S31. GPC graphic of **$CF_3Ph^FPh^F$-Se-PMS**.

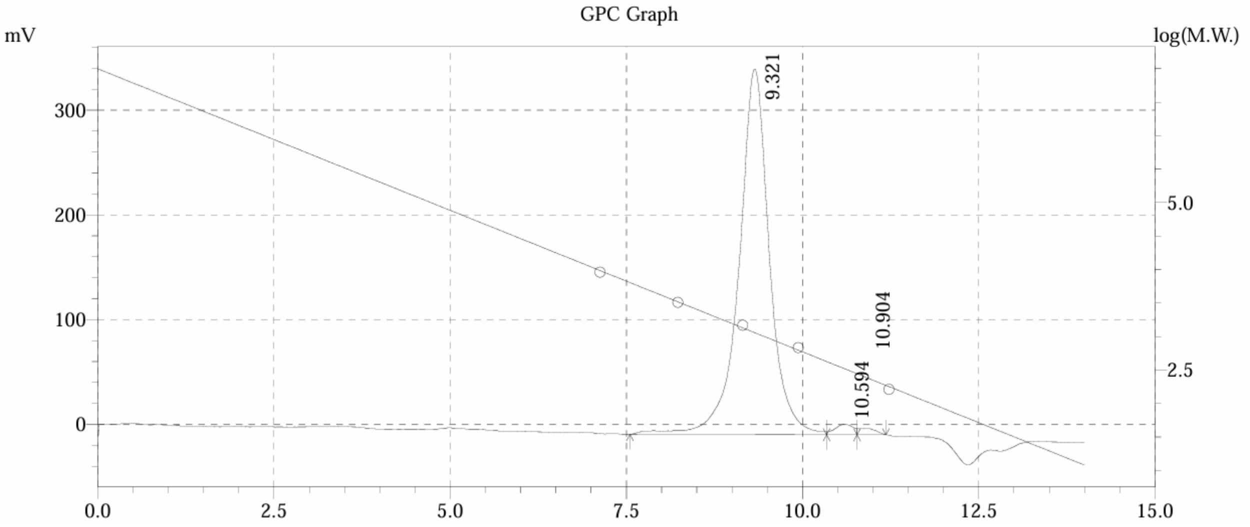

Figure S32. GPC graphic of **$PhPh^F$-Se-PMS**.

S4. TGA data

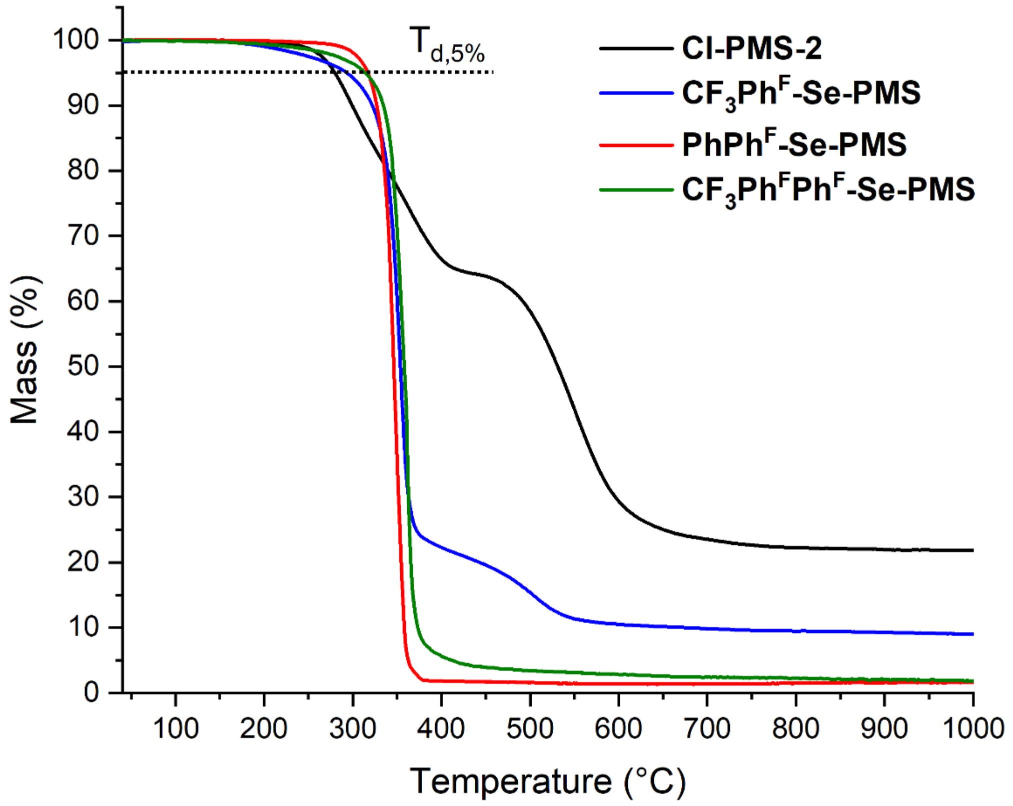

Figure S33. TGA curves of synthesized polymers. $T_{d,5\%}$: **Cl-PMS** – 280 °C, **PhPh^F-Se-PMS** – 317 °C, **CF$_3$Ph^F-Se-PMS** – 293 °C, **CF$_3$Ph^{F}Ph^F-Se-PMS** – 314 °C.

S5. Hydrophobicity test

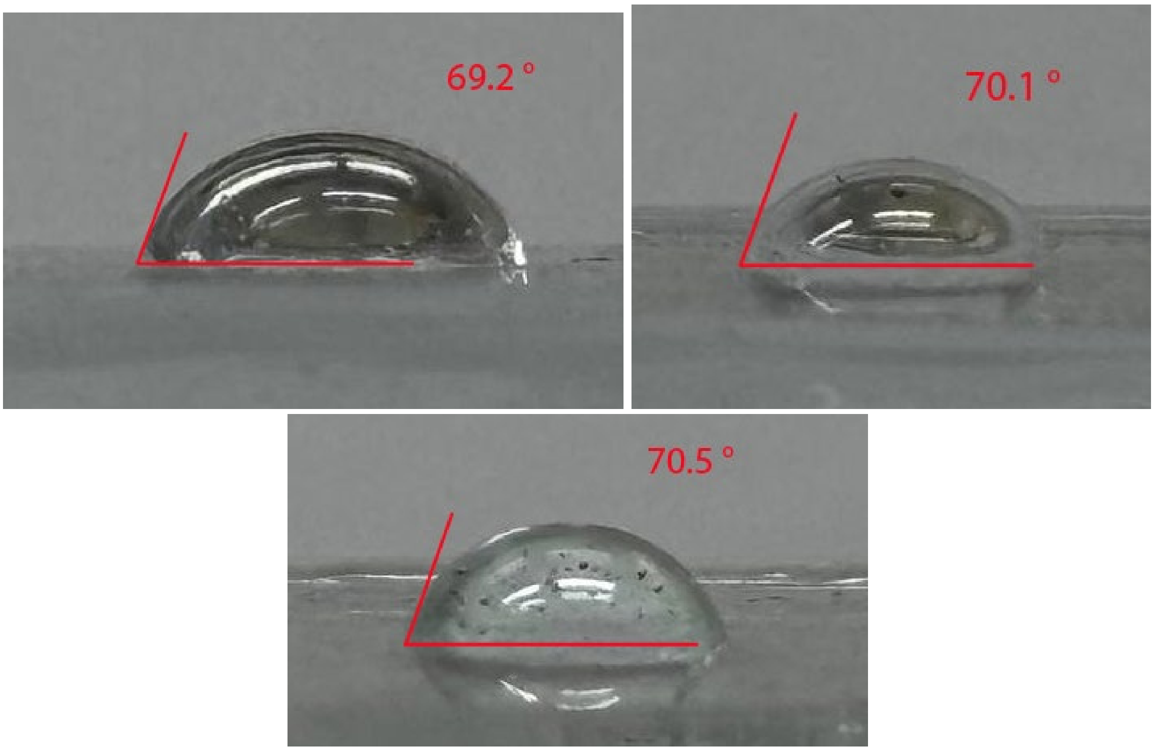

Figure S34. Representative contact angles for **Cl-PMS**.

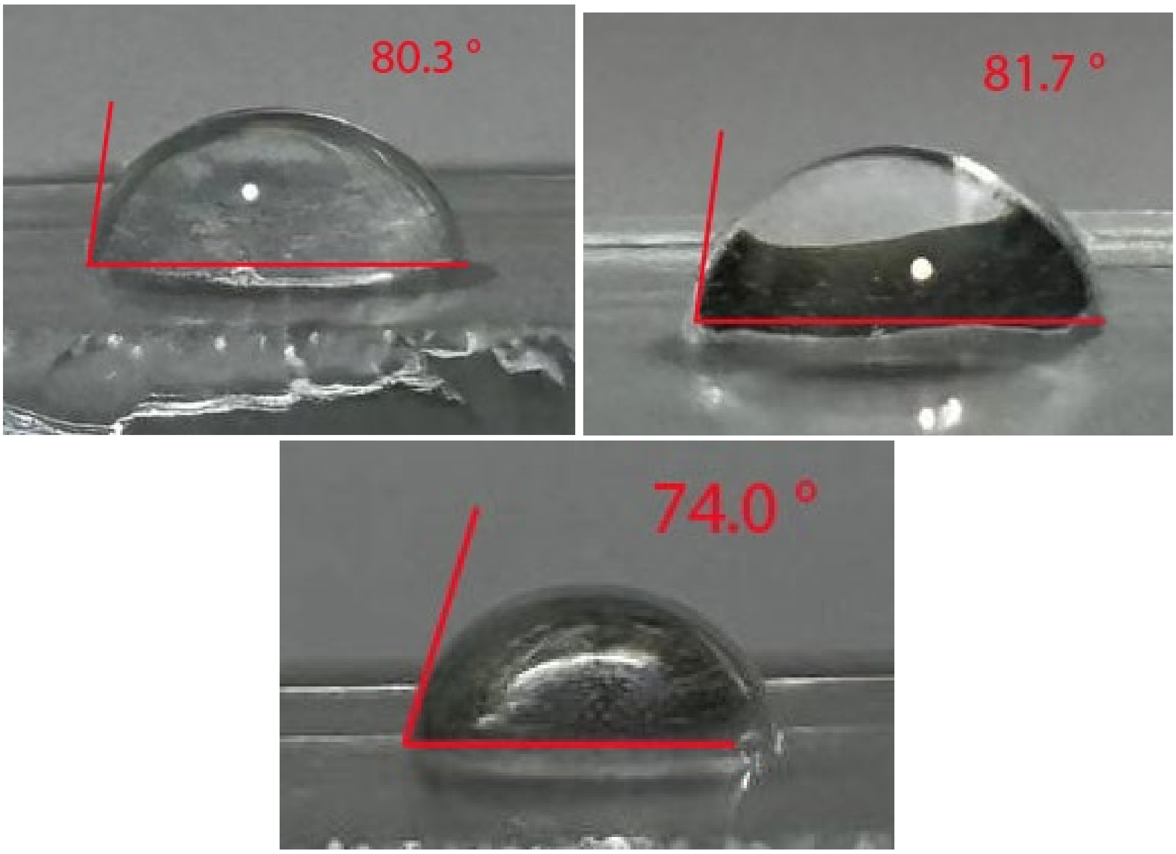

Figure S35. Representative contact angles for **CF_3Ph^F-Se-PMS**.

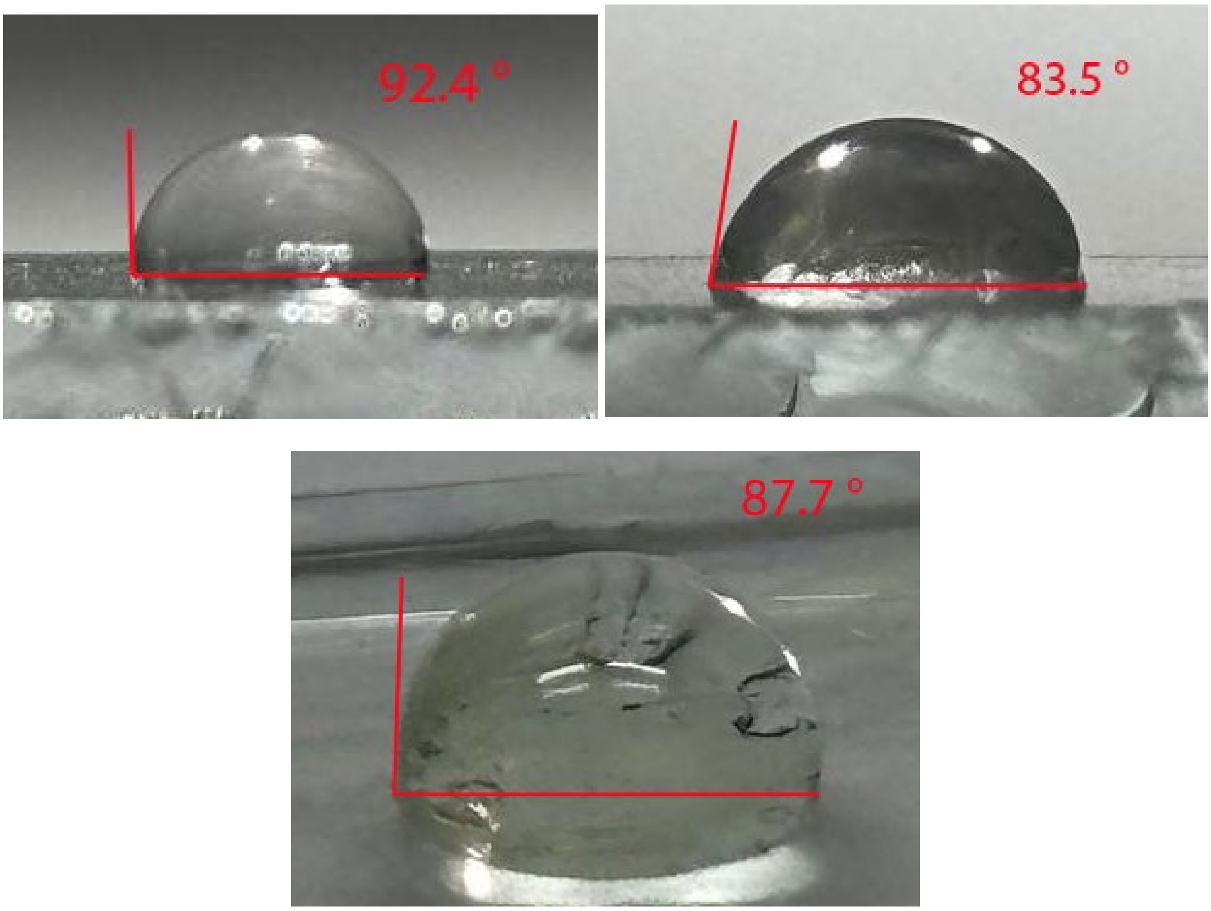

Figure S36. Representative contact angles for **PhPh^{F}-Se-PMS**.

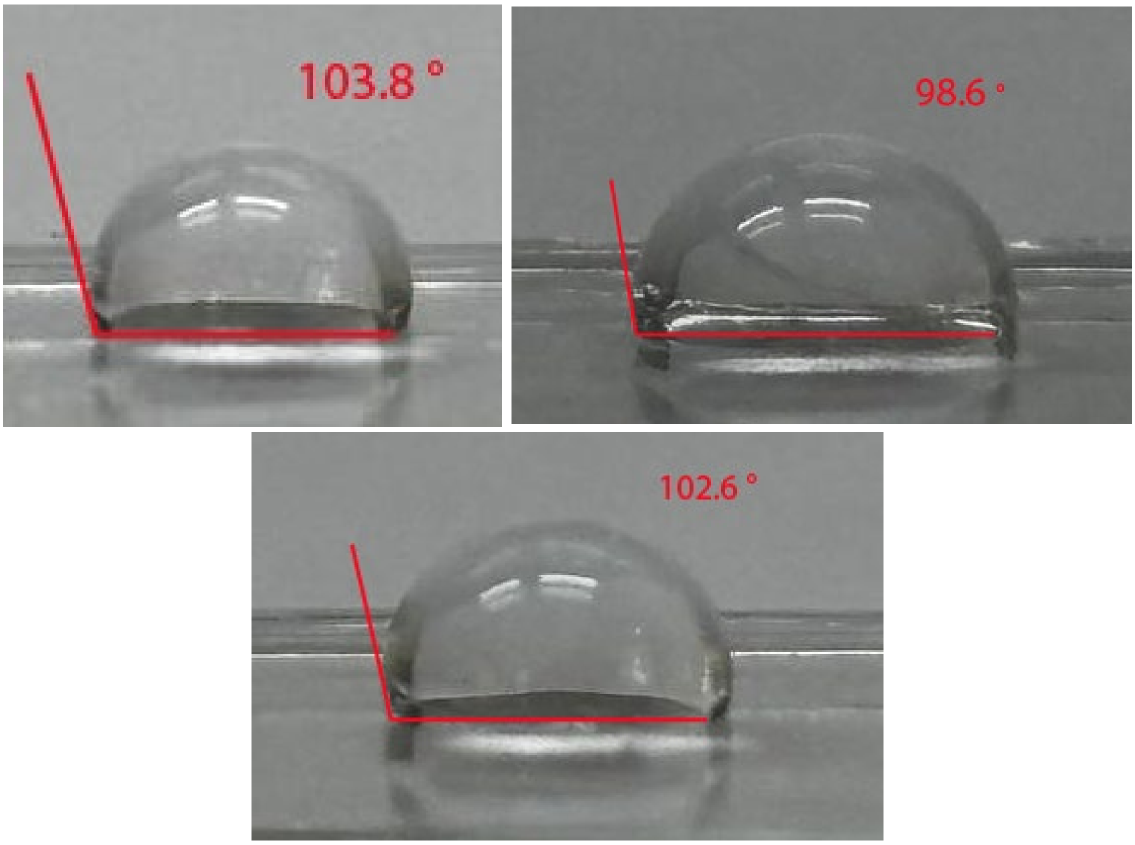

Figure S37. Representative contact angles for **CF$_3$Ph^{F}Ph^{F}-Se-PMS**.
